# Low-Energy Light-Driven Excited-State Palladium Catalysis: Cross-Coupling of Pyridine *N*‑Oxides with Unactivated Alkyl Bromides

**DOI:** 10.1021/acs.orglett.6c01179

**Published:** 2026-05-08

**Authors:** Jisun Kim, Minyoung Ju, Chaerin Baek, Eun Joo Roh, Jeongcheol Shin, Jung Tae Han

**Affiliations:** † Chemical and Biological Integrative Research Center, Korea Institute of Science and Technology (KIST), 02792 Seoul, Republic of Korea; ‡ Division of Bio-Medical Science and Technology, KIST School, University of Science and Technology (UST), 02792 Seoul, Republic of Korea; § Department of Chemistry, 34945 Duksung Women’s University, 01369 Seoul, Republic of Korea

## Abstract

Excited-state Pd catalysis has emerged as a new platform for cross-coupling reactions. Nonetheless, such catalysis typically requires high-energy near-ultraviolet (NUV) or blue (380–450 nm) light. In contrast, the use of lower-energy light (>520 nm) in excited-state Pd catalysis remains largely unexplored. Herein, we report a low-energy light-driven excited-state Pd-catalyzed cross-coupling of pyridine *N*-oxides with unactivated alkyl bromides. Mechanistic studies reveal the formation of a Pd(0)–pyridine *N*-oxide complex that absorbs low-energy light.

## Graphical abstract

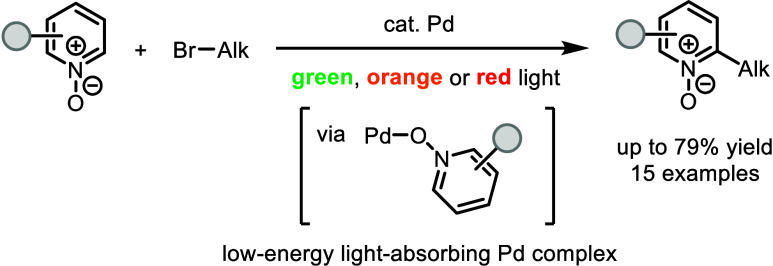

Pd-catalyzed cross-coupling reactions are fundamental transformations in organic synthesis, enabling the construction of C–C, C–N, C–O, C–X, and other C–heteroatom bonds.^1^ Traditionally, these reactions are initiated by ground-state Pd catalysts and proceed through two-electron redox processes. Recently, excited-state Pd catalysts have emerged and found applications in cross-coupling reactions that proceed through single-electron transfer (SET) processes.^2^ Representative examples include the Mizoroki–Heck reaction,^3^ the Negishi reaction,^4^ the Suzuki–Miyaura reaction,^5^ Miyaura borylation,^6^ carbonylation,^7^ and C–H functionalization.^8^ However, these reactions typically require high-energy near-ultraviolet (NUV) or blue light (380–450 nm) and thus suffer from issues such as limited scalability, functional group incompatibility, and competitive photoexcitation of substrates (Scheme 1a).^9^ Alternatively, the use of lower-energy green, orange, or red light (520–740 nm) could address the issues associated with NUV or blue light;^10^ however, such light lacks the energy to excite the ground-state Pd precatalyst (Scheme 1b).^11^ Despite this significant challenge, Iwasawa has recently demonstrated that low-energy light-driven excited-state Pd catalyzes a C–O cross-coupling reaction, albeit with only a single example in which 4-*tert*-butyl iodobenzene is coupled with cesium pivalate (Scheme 1c).^12^

**Scheme 1:**
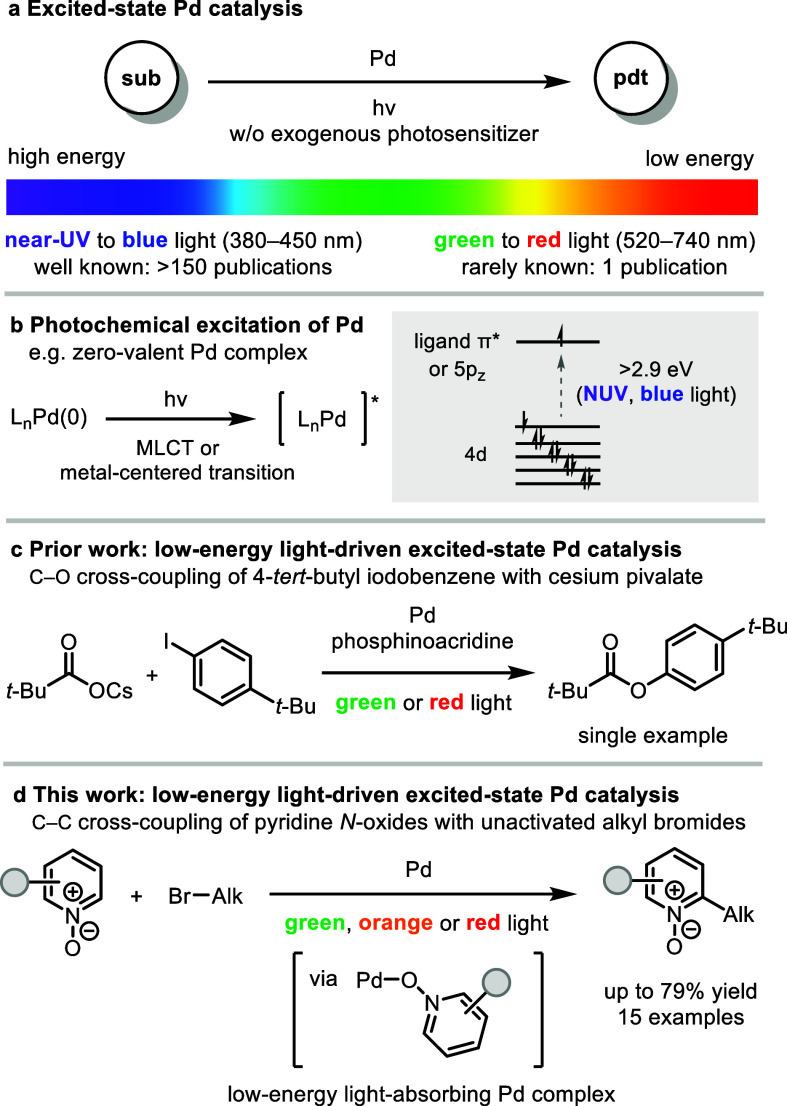
Overview of Excited-State Pd Catalysis

Alkylation of pyridine *N*-oxides provides efficient access to alkylated pyridine *N*-oxides and pyridines, which are found in many biologically active molecules and pharmaceuticals.^13^ Thus, numerous catalytic and stoichiometric methods have been developed for the alkylation of pyridine *N*-oxides.^14^ However, only a few methods employing Pd catalysts have been reported, despite significant advances in Pd-catalyzed cross-coupling reactions. In 2013 and 2014, Fu and Zhou independently reported Pd-catalyzed cross-coupling of pyridine *N*-oxides with unactivated alkyl bromides and iodides under thermal conditions (100–110 °C).^15^ More recently, Yu and Fu independently demonstrated that blue light-driven excited-state Pd catalyzes the same transformation, although they reported only a single example in which 2-methylpyridine *N*-oxide reacts with 1-adamantyl bromide.^8a,8c^

Inspired by prior work by Iwasawa,^12^ we hypothesized that in the presence of an appropriate ligand, a ground-state Pd precatalyst could be photoexcited by low-energy light and the resulting excited-state Pd catalyst could promote the C–C cross-coupling reaction. We herein report a low-energy light-driven excited-state Pd-catalyzed cross-coupling of pyridine *N*-oxides with unactivated alkyl bromides (Scheme 1d). Mechanistic studies reveal that pyridine *N*-oxide substrates act as ligands to Pd(0), forming Pd(0)–pyridine *N*-oxide complexes that absorb low-energy light.

Our investigation began with the cross-coupling of 2-methylpyridine *N*-oxide (**1a**) with cyclohexyl bromide (**2a**) (Table 1). Following an extensive study of reaction parameters, we found that a combination of Pd­(PPh_3_)_4_ (10 mol %), Cs_2_CO_3_ (1.5 equiv), and TBAB (1.5 equiv) in THF at 25 °C under green light (525 nm) irradiation *without an external ligand* provides product **3aa** in 60% yield (entry 1). The use of Pd­(OAc)_2_ in conjunction with PPh_3_ slightly decreased the yield of **3aa** (entry 2); the use of Pd­(PPh_3_)_2_Cl_2_ delivered **3aa** in only 8% yield (entry 3). Notably, external ligands (BINAP, Xantphos, DPEphos, and DPPF) resulted in lower yields (entries 4–7, respectively). Amine bases such as diisopropylethylamine (DIPEA) showed reduced performance (entry 8). Altering the light sources led to either decreased yields or no product formation (entries 9–12). Whereas cyclohexyl iodide afforded a yield comparable to that of cyclohexyl bromide (**2a**), cyclohexyl chloride was shown to be unreactive (entries 13 and 14). In the absence of Pd­(PPh_3_)_4_ or light, **3aa** was not formed (entry 15 or 16, respectively); in the absence of TBAB, which has been shown to enhance the reactivity in blue light-driven excited-state Pd catalysis,^8e^ the yield of **3aa** decreased slightly (entry 17). The role of TBAB in this transformation is unclear at this time. When the reaction was conducted under an ambient atmosphere, no product was formed, presumably due to quenching of the active Pd catalyst by oxygen (entry 18). Lastly, increasing the Pd­(PPh_3_)_4_ loading to 15 mol % allowed product **3aa** to be obtained in 75% yield (entry 19).

**Table 1:**
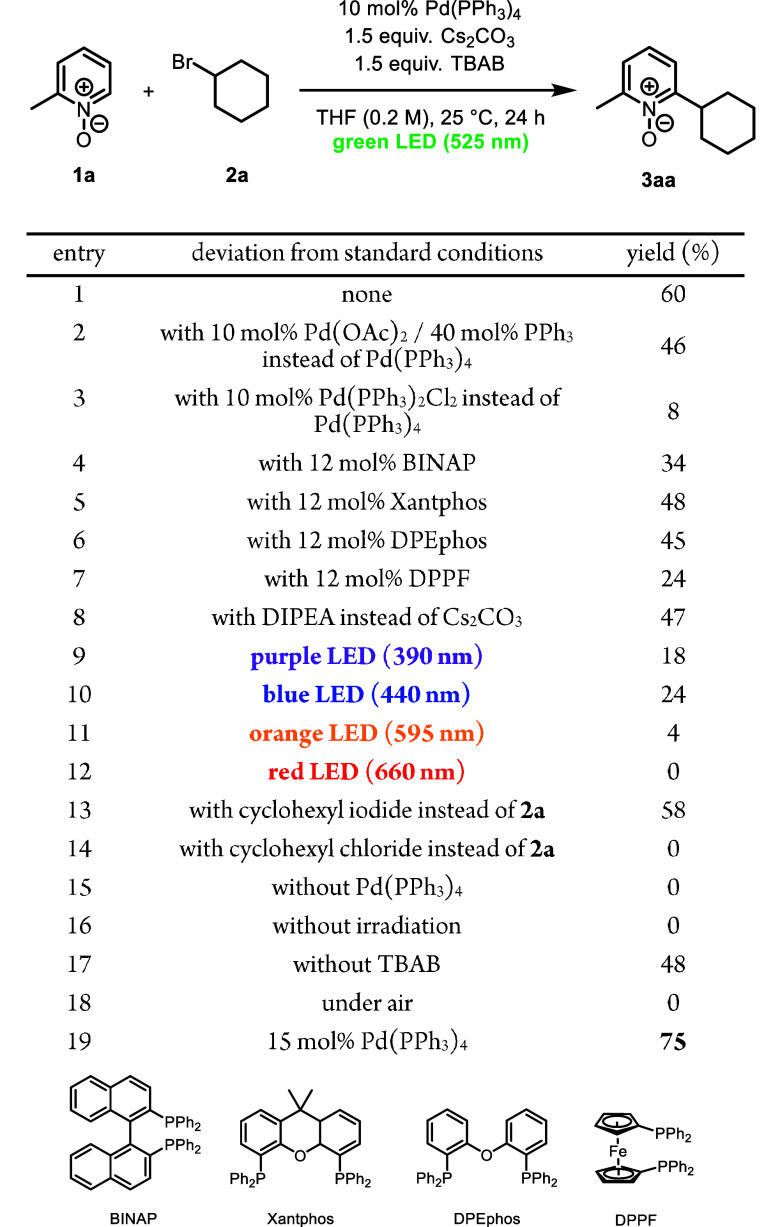
Optimization^a^,^b^

a Reactions were conducted on a 0.2 mmol scale.

b Yields were determined by ^1^H NMR analysis of the unpurified reaction mixture with dibromomethane as an internal standard.

With the optimized reaction conditions in hand, we investigated the scope of unactivated alkyl bromides (Scheme 2). Cyclic and acyclic secondary alkyl bromides reacted efficiently to provide the desired products (**3aa**–**3ag**). Remarkably, when *exo*-2-bromonorbornane (**2e**) was employed, complete diastereoselectivity was observed. In contrast to secondary alkyl bromides, primary and tertiary alkyl bromides tended to furnish the products in lower yields (**3ah** and **3ai**). We next examined the scope of pyridine *N*-oxides. 2-Phenylpyridine *N*-oxide (**1b**) was also a suitable substrate for the transformation, but in this case, 1,4-dioxane was needed as the solvent. When quinoline *N*-oxide (**1c**) was subjected to the optimized reaction conditions, only a trace of the desired product was observed. After extensive reoptimization, we were pleased to find that the reaction required BINAP as an external ligand to provide the desired product in 76% yield. Under these modified reaction conditions, quinoline *N*-oxide derivatives (**1d**–**1g**) were transformed into the corresponding products in satisfactory yields (70–96%). Finally, we sought to use lower-energy light in our catalyst system. Under orange and red light irradiation, quinoline *N*-oxide (**1c**), lepidine *N*-oxide (**1d**), and benzo­[*h*]­quinoline *N*-oxide (**1g**) were all reactive. These results are notable because low-energy orange or red light-driven excited-state transition-metal catalysis has rarely been reported.^16^ Finally, an 8 mmol scale reaction was conducted, and 0.93 g of product **3aa** was obtained in 61% yield (see the Supporting Information for details).

**Scheme 2:**
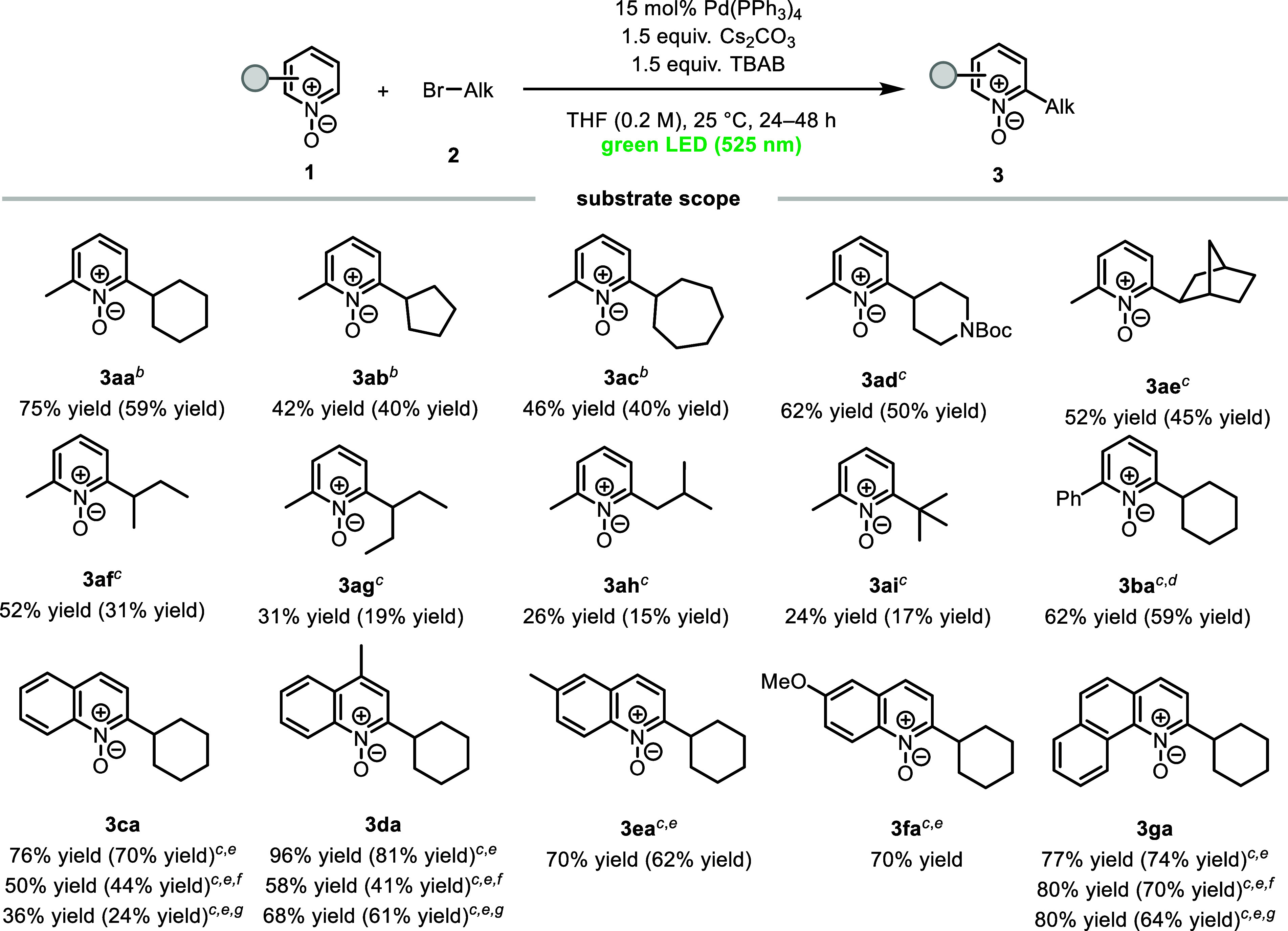
Substrate Scope^a^

a Reactions were conducted on a 0.2 mmol scale. Yields were determined by ^1^H NMR analysis of the unpurified reaction mixture with dibromomethane as an internal standard. Yields in parentheses are those of isolated products.
b For 24 h.
c For 48 h.
d 1,4-Dioxane was used as a solvent instead of THF.
e With 15 mol % BINAP used as a ligand.
f The reaction was conducted under an orange LED (595 nm).
g The reaction was conducted under a red LED (660 nm).

To gain mechanistic insights into the low-energy light-driven excited-state Pd-catalyzed cross-coupling reaction, we carried out radical trapping and control experiments (Scheme 3). When 2,2,6,6-tetramethylpiperidin-1-oxyl (TEMPO), a radical trap, was subjected to the standard reaction conditions, the formation of product **3aa** was inhibited and TEMPO adduct **4** was detected by electrospray ionization mass spectrometry (ESI-MS) (Scheme 3a). This is consistent with our hypothesis that the reaction involves alkyl radical intermediates. Under otherwise identical conditions in the absence of **1a**, TEMPO adduct **4** was not formed; however, upon blue light irradiation, TEMPO adduct **4** was detected (Scheme 3b). These results indicate that **1a** is essential for the low-energy light-driven Pd-catalyzed cross-coupling reaction and suggest the formation of a putative Pd(0)·**1a** complex that absorbs low-energy light.

**Scheme 3:**
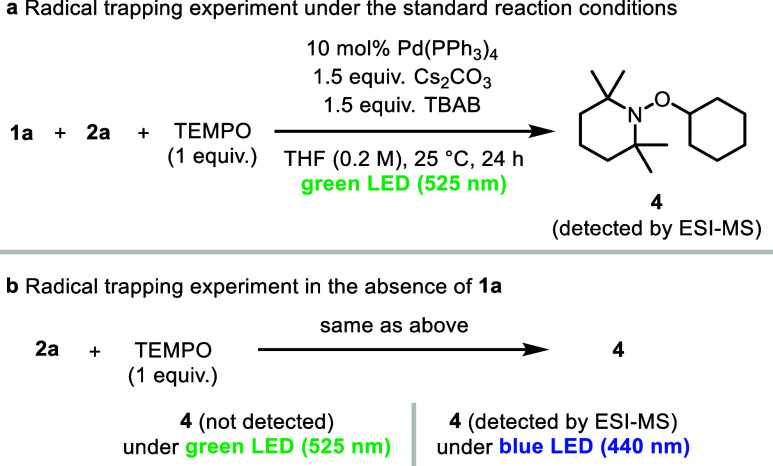
Radical Trapping and Control Experiments

Next, we investigated the formation of the Pd(0)·**1a** complex by UV–vis–NIR absorption spectroscopy (Figure 1).^17^ None of the individual reaction components [**1a**, **2a**, Pd­(PPh_3_)_4_, DIPEA, and TBAB] absorbed at around the operational wavelength (525 nm) (Figure 1a). In contrast, upon mixing Pd­(PPh_3_)_4_ with 20 equiv of **1a**, a diagnostic absorption at 505 nm was observed, providing evidence that the Pd(0)·**1a** complex absorbs green light (Figure 1b). However, addition of **2a**, DIPEA, and TBAB resulted in an only negligible bathochromic shift. To assign the observed absorption, time-dependent density functional theory (TD-DFT) calculations were conducted. First, the absorption spectra of Pd­(PPh_3_)_4_, Pd­(PPh_3_)_3_, and Pd­(PPh_3_)_2_ were simulated, as PPh_3_ is known to reversibly dissociate from Pd­(PPh_3_)_4_ in THF.^18^ However, none of these species exhibited absorbance above 500 nm (Figure 1c). Further calculations revealed that Pd­(PPh_3_)_4_ and **1a** associate to form a linear Pd­(PPh_3_)·**1a** complex with a highly exergonic Δ*G* (−16.6 kcal/mol) (Figure 1d,e). Importantly, the simulated absorption spectrum of the Pd­(PPh_3_)·**1a** complex was in close agreement with the experimental spectrum (Figures 1b,c). The possibility that the Pd­(PPh_3_)_3_·**1a** or Pd­(PPh_3_)_2_·**1a** complex is the green light-absorbing species was ruled out, as geometry optimization resulted in spontaneous dissociation of **1a** from the Pd complex. Frontier molecular orbital (FMO) analysis of the Pd­(PPh_3_)·**1a** complex suggests that the low-energy light absorption corresponds primarily to a metal-to-ligand charge transfer (MLCT) from the Pd 4d_*z* ^2^_-based MO to the **1a** π*-based MO (Figure 1f).^19^ The resulting excited-state species, Pd­(I)­(PPh_3_)­(**1a** ^•–^), could promote subsequent steps such as halogen atom transfer (XAT) of **2a** or C–H activation of **1a**.

**Figure 1:**
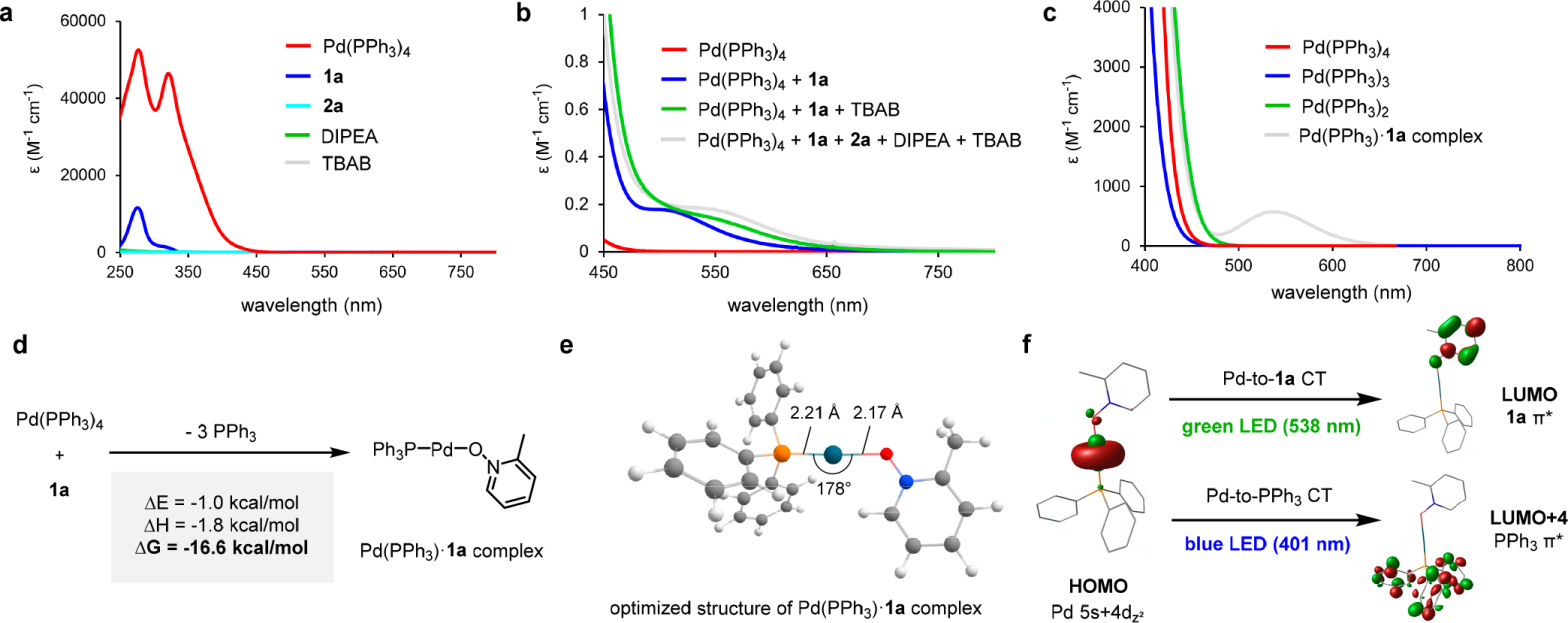
Experimental absorption spectra of (a) individual reaction components and (b) their mixture. (c) TD-DFT-simulated absorption spectra of Pd complexes. DFT-calculated (d) formation thermodynamics and (e) geometry of the Pd­(PPh_3_)·**1a** complex. (f) Frontier molecular orbital (FMO) analysis of the Pd­(PPh_3_)·**1a** complex.

On the basis of radical trapping and control experiments, UV–vis–NIR absorption studies, and TD-DFT calculations along with generally accepted mechanisms for excited-state Pd catalysis,^2,8a,15b,20^ a catalytic cycle for this transformation is proposed (Scheme 4). The association of Pd(0) with pyridine *N*-oxide **1** forms Pd(0)–pyridine *N*-oxide complex **I**, which is photoexcited to I* under low-energy light irradiation. Subsequent XAT of unactivated alkyl bromide **2** to I* generates hybrid Pd­(I) alkyl radical **II**. Radical insertion of **II** into **1** then affords hybrid Pd­(I) nitrogen-centered radical **III**. Finally, single-electron transfer of **III** followed by deprotonation provides product **3** and regenerates **I**. Alternative mechanisms such as preferential C–H activation of **1** with I* over the XAT process cannot be ruled out at this time.

**Scheme 4:**
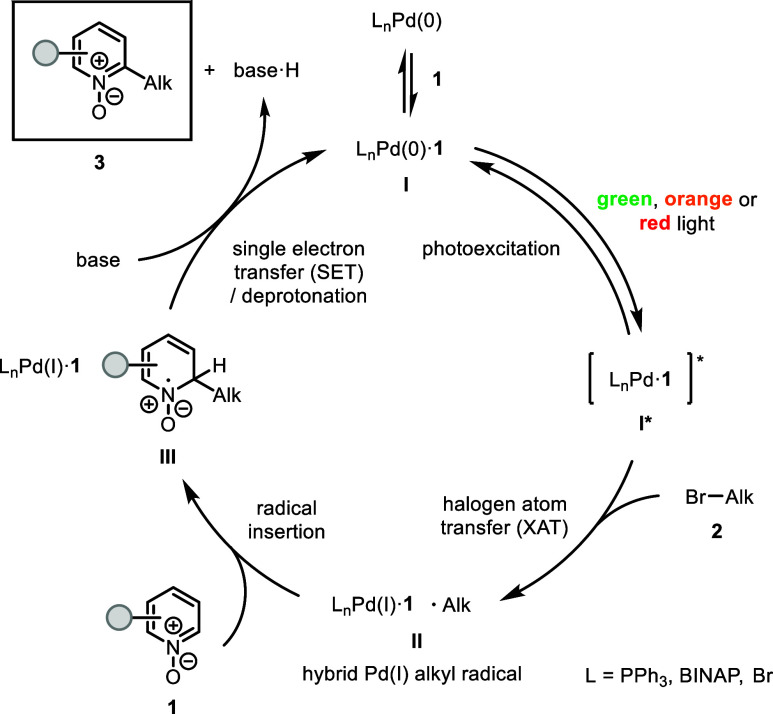
Proposed Catalytic Cycle

In conclusion, we have developed a low-energy light-driven excited-state Pd-catalyzed cross-coupling of pyridine *N*-oxides with unactivated alkyl bromides. This catalytic protocol exhibits a broad substrate scope, providing alkylated pyridine *N*-oxides in moderate to high yields. Mechanistic studies reveal that the reaction proceeds through the formation of a low-energy light-absorbing Pd(0)–pyridine *N*-oxide complex. The isolation and characterization of this complex are the subjects of ongoing research in our laboratory.

## Supplementary Material



## Data Availability

The data underlying this study are available in the published article and its Supporting Information.

## Notes

Experimental procedures and NMR spectra of the products (PDF)
