# Supplementary material for: Low-Energy Light-Driven Excited-State Palladium Catalysis: Cross-Coupling of Pyridine N‑Oxides with Unactivated Alkyl Bromides
Source: Org Lett. 2026 May 8;28(20):6208–13. doi: 10.1021/acs.orglett.6c01179 (PMC13200257; doi:10.1021/acs.orglett.6c01179)
Supplement: Supplementary file 1 [file ol6c01179_si_001.pdf]

Supplementary Information

Low-Energy Light-Driven Excited-State Palladium Catalysis: Cross-Coupling of Pyridine *N*-Oxides with Unactivated Alkyl Bromides

Jisun Kim[1,2], Minyoung Ju[3], Chaerin Baek,[3] Eun Joo Roh[1,2], Jeongcheol Shin[3*], Jung Tae Han[1,2]*

[1]Chemial and Biological Integrative Research Center, Korea Institute of Science and Technology (KIST), Seoul 02792, Republic of Korea

[2]Division of Bio-Medical Science and Technology, KIST School, University of Science and Technology (UST), Seoul 02792, Republic of Korea

[3]Department of Chemistry, Duksung Women's University, Seoul 01369, Republic of Korea

*Email: jcshin91@duksung.ac.kr; jungtae@kist.re.kr

Content

1. General considerations S2

2. Optimization S3

3. General procedure for the cross-coupling of pyridine *N*-oxides with unactivated alkyl bromides S5

4. Analytic data of products S5

5. 8 mmol scale reaction S10

6. Limitations S10

7. Radical trapping and control experiments S12

8. UV-Vis-NIR absorption studies S14

9. Titration experiments S14

10. DFT calculations S16

11. References S21

12. Copies of NMR spectra S22

1. General considerations

Chemicals

Unless otherwise noted, starting materials and anhydrous solvents were obtained from Sigma-Aldrich or TCI. Moreover, commercially available reagents were used without additional purification. The pyridine *N*-oxides **1b**, **1d**, **1e**, **1g** were synthesized according to literature procedures.[1]

Light sources

40W Kessil PR160L LEDs (390, 440, 525, 595, and 660 nm) were used as light sources for the reactions at maximum intensity. The emission spectra of the 390, 440, and 525 nm LEDs are available at https://kessil.com/products/science_PR160L.php; the emission spectra of the 595 and 660 nm LEDs have been reported in the literatures.[2,3]

Inert Gas

Dry argon was purchased from Shinyang Medicine with >99.9% purity.

Thin Layer Chromatography

Thin-layer chromatography (TLC) was performed using silica gel coated glass sheets (TLC Silica gel 60 F_{254}, 0.2 mm, with fluorescent indicator; Merck) which was visualized with a UV lamp (254 nm) and/or basic $KMnO_4$.

Column Chromatography

Column chromatography (CC) was carried out using Merck silica gel (60 Å, 230−400 mesh, particle size 0.040−0.063 mm) using technical grade solvents. Elution was accelerated using compressed argon. All reported yields, unless otherwise noted, refer to spectroscopically and chromatographically pure compounds.

Nomenclature

Nomenclature follows the suggestions proposed by the computer program ChemDraw (23.1.2.7) of Revvity Signals Software.

Nuclear Magnetic Resonance Spectroscopy

^{1}H, ^{13}C Nuclear magnetic resonance (NMR) spectra for compound characterization were recorded on Bruker AVIII-400 MHz, NMR spectrometer in a suitable deuterated solvent. The solvent employed and the respective measuring frequency are indicated for each experiment. Chemical shifts are reported with tetramethylsilane (TMS) serving as a universal reference of all nuclides. The resonance multiplicity is described as s (singlet), d (doublet), t (triplet), q (quadruplet), m (multiplet), and b (broad). All spectra were recorded at 298 K, processed with MestReNova 15.1.0 suits of program, and coupling constants are reported as observed. The residual deuterated solvent signal relative to tetramethylsilane was used as the internal reference in ^{1}H NMR spectra (e.g. $CDCl_3$ = 7.26 ppm, CD_2Cl_2 = 5.32 ppm). Signals are reported as follows: chemical shift δ in ppm (multiplicity, coupling constant *J* in Hz, number of protons). All X-nuclei spectra were acquired proton decoupled unless otherwise noted.

Mass Spectrometry

Electrospray ionization (ESI) mass spectrometry was conducted on a Xevo G2-XS QTOF spectrometer (Waters, Milford, MA, USA) at the Chiral Material Core Facility Center of Sungkyunkwan University. The ionization method and mode of detection employed is indicated for the respective experiment and all masses are reported in atomic units per elementary charge (*m/z*) with an intensity normalized to the most intense peak.

Abbreviations

TLC = thin layer chromatography, THF = tetrahydrofuran, DMF = *N*,*N*-dimethylformamide, DMA = *N*,*N*-dimethylacetamide, DMSO = dimethylsulfoxide, DMI = *N*,*N*-dimethylimidazolidinone, NMP = *N*-methylpyrrolidone, DME = 1,2-dimethoxyethane, TBAB = tetrabutylammonium bromide, TBAC = tetrabutylammonium chloride, TBAI = tetrabutylammonium iodide, BINAP = 2,2′-bis(diphenylphosphino)-1,1′-binaphthalene, Xantphos = 4,5-bis(diphenylphosphino)-9,9-dimethylxanthene, DPEphos = bis[(2-diphenylphosphino)phenyl] ether, DPPF = 1,1′-ferrocenediyl-bis(diphenylphosphine).

2. Optimization

Table S1. Solvent effect[a]

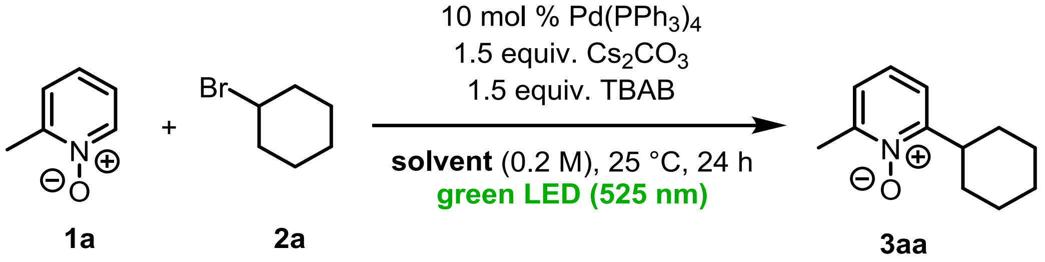

entry	solvent	yield
**1**	**THF**	**60**
2	THF:H_2O (10:1)	40
3	DMA	58
4	MeCN	21
5	DMSO	37
6	2-Me-THF	53
7	PhMe	47
8	1,4-dioxane	59
9	DMI	41
10	DMF	47
11	NMP	46
12	DME	42
13	EtOAc	57
14	CH_2Cl_2	0

[a]Reactions were conducted on 0.2 mmol scale. Yields were determined by ^{1}H NMR analysis with dibromomethane as an internal standard.

Table S2. Pd precursor effect.[a]

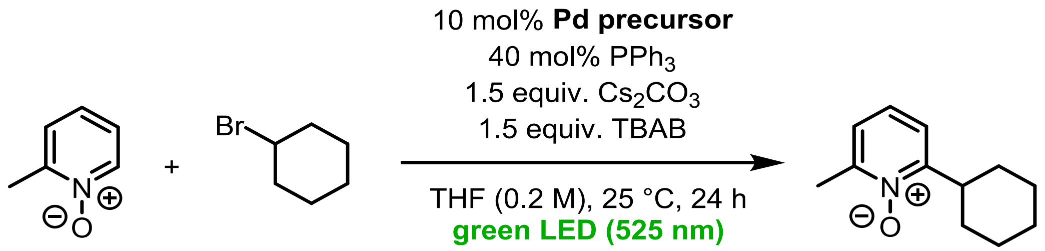

1a **2a** **3aa**

entry	Pd precursor	yield
1	$Pd(PPh_3)_4$ w/o PPh_3	60
2	$Pd(OAc)_2$	46
3	$Pd(TFA)_2$	48
4	$PdCl_2$	45
5	$Pd_2(dba)_3$	0
6	Pd(COD)(DQ)	0

[a]Reactions were conducted on 0.2 mmol scale. Yields were determined by ^{1}H NMR analysis with dibromomethane as an internal standard.

Table S3. Additive effect.[a]

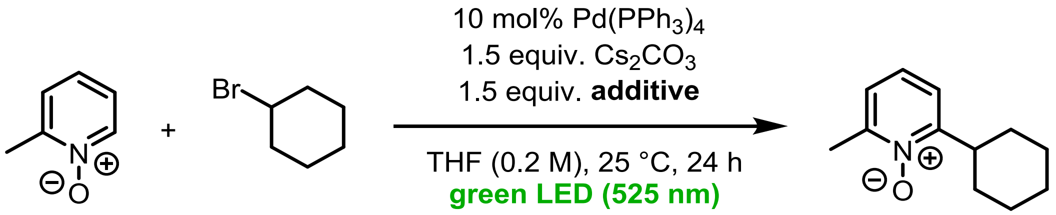

1a **2a** **3aa**

entry	additive	yield
**1**	**TBAB**	**60**
2	TBAC	38
3	TBAI	38

[a]Reactions were conducted on 0.2 mmol scale. Yields were determined by ^{1}H NMR analysis with dibromomethane as an internal standard.

3. General procedure for the cross-coupling of pyridine *N*-oxides with unactivated alkyl bromides

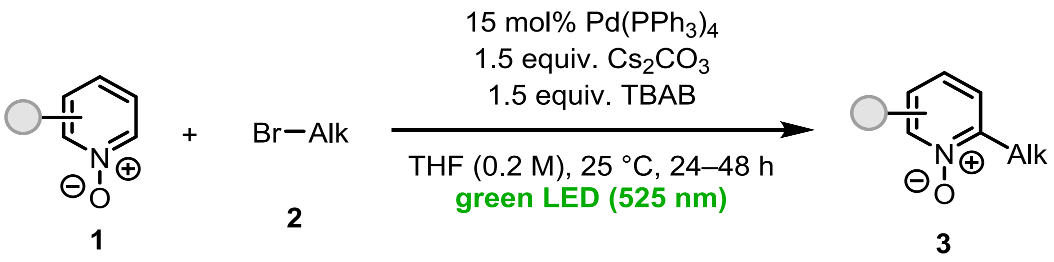

A flame-dried Schlenk tube equipped with a magnetic bar was charged with $Pd(PPh_3)_4$ (0.03 mmol, 15 mol%), Cs_2CO_3 (0.3 mmol, 1.5 equiv.) and TBAB (0.3 mmol, 1.5 equiv.). The tube was evacuated and backfilled with nitrogen; this process was repeated three times. Pyridine *N*-oxide (0.4 mmol, 2 equiv.) as a solution in THF (1 mL, 0.2 M) and alkyl bromide (0.2 mmol) were added. The Schlenk tube was capped and allowed to stir under green LED irradiation (40W, 525 nm) with fan cooling (reaction temperature: 25 °C; distance between the LED and the Schlenk tube: 0.4 cm). After 24–48 h, the reaction mixture was transferred to a 100 mL round bottom flask and the solvent was removed *in vacuo*. H_2O (10 mL) was added and the mixture was extracted with EtOAc (3 x 10 mL). The combined organic layers were washed with brine, dried over Na_2SO_4, filtered, and concentrated *in vacuo*. Dibromomethane was added as an internal standard to determine the NMR yield. The mixture was purified by column chromatography on silica gel (hexane/acetone) to afford the desired product.

4. Analytic data of products

2-cyclohexyl-6-methylpyridine 1-oxide (3aa)

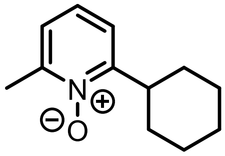

The reaction was conducted on a 0.2 mmol scale. The titled product was purified by column chromatography (hexane/acetone) to afford **3aa** as a colorless oil (22.6 mg, 59% yield)

^{1}H NMR (400 MHz, $CDCl_3$) δ 7.18–7.02 (m, 3H), 3.56 (t, *J* = 2.8 Hz, 1H), 2.52 (s, 3H), 2.04 (d, *J* = 11.4 Hz, 2H), 1.89–1.76 (m, 3H), 1.51 (dd, *J* = 13.0, 3.0 Hz, 2H), 1.27 (ddd, *J* = 15.1, 7.3, 3.0 Hz, 3H).

^{13}C NMR (101 MHz, $CDCl_3$) δ 156.9, 149.2, 125.2, 123.5, 120.7, 37.7, 31.2, 26.6, 26.4, 18.7.

These spectroscopic data are consistent with those previously reported in the literature.[4]

2-cyclopentyl-6-methylpyridine 1-oxide (3ab)

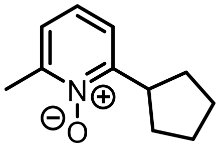

The reaction was conducted on a 0.2 mmol scale. The titled product was purified by column chromatography (hexane/acetone) to afford **3ab** as a colorless oil (14.2 mg, 40% yield)

^{1}H NMR (400 MHz, $CDCl_3$) δ 7.20–7.06 (m, 3H), 3.81 (p, *J* = 8.4 Hz, 1H), 2.54 (s, 3H), 2.26–2.17 (m, 2H), 1.82–1.69 (m, H), 1.61–1.50 (m, 2H).

^{13}C NMR (101 MHz, $CDCl_3$) δ 156.3, 149.3, 124.8, 123.6, 120.7, 39.8, 31.2, 25.5, 18.7.

These spectroscopic data are consistent with those previously reported in the literature.[4]

2-cycloheptyl-6-methylpyridine 1-oxide (3ac)

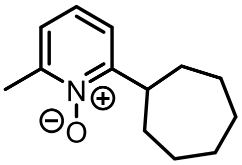

The reaction was conducted on a 0.2 mmol scale. The titled product was purified by column chromatography (hexane/acetone) to afford **3ac** as a colorless oil (16.4 mg, 40% yield)

^{1}H NMR (400 MHz, $CDCl_3$) δ 7.10 (s, 3H), 3.77–3.60 (m, 1H), 2.52 (s, 3H), 2.05−1.94 (m, 2H), 1.83−1.49 (m, 10H).

^{13}C NMR (101 MHz, $CDCl_3$) δ 158.4, 149.1, 125.0, 123.3, 120.8, 38.8, 33.5, 27.9, 27.4, 18.7.

These spectroscopic data are consistent with those previously reported in the literature.[4]

2-(1-(*tert*-butoxycarbonyl)piperidin-4-yl)-6-methylpyridine1-oxide (3ad)

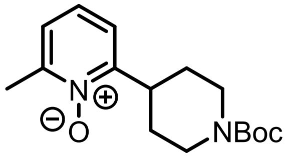

The reaction was conducted on a 0.2 mmol scale. The titled product was purified by column chromatography (hexane/acetone) to afford **3ad** as a colorless oil (29.2 mg, 50% yield)

^{1}H NMR (400 MHz, $CDCl_3$) δ 7.21−7.10 (m, 2H), 7.09−7.03 (m, *J* = 6.4 Hz, 1H), 4.27 (s, 2H), 3.71 (t, *J* = 12.0 Hz, 1H), 2.90 (s, 2H), 2.53 (s, 3H), 2.04 (d, *J* = 12.4 Hz, 2H), 1.47 (s, 11H).

^{13}C NMR (101 MHz, $CDCl_3$) δ 154.9, 154.7, 149.3, 125.1, 124.1, 120.6, 79.7, 43.9, 36.2, 29.8, 28.6, 18.6.

These spectroscopic data are consistent with those previously reported in the literature.[4]

2-((1*S*,2*S*,4*R*)-bicyclo[2.2.1]heptan-2-yl)-6-methylpyridine 1-oxide (3ae)

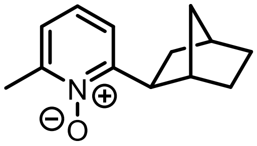

The reaction was conducted on a 0.2 mmol scale. The titled product was purified by column chromatography (hexane/acetone) to afford **3ae** as a colorless oil (17.0 mg, 45% yield)

^{1}H NMR (400 MHz, $CDCl_3$) δ 7.20−7.02 (m, 3H), 3.43−3.29 (m, 1H), 2.51 (s, 3H), 2.44 (s, 1H), 2.35 (s, 1H), 2.05 (ddd, *J* = 11.8, 9.0, 2.2 Hz, 1H), 1.70−1.54 (m, 2H), 1.53−1.34 (m, 4H), 1.34−1.16 (m, 1H).

^{13}C NMR (101 MHz, $CDCl_3$) δ 156.6, 149.2, 124.5, 123.3, 120.3, 42.2, 40.1, 37.4, 36.9, 36.8, 30.1, 29.0, 18.6.

These spectroscopic data are consistent with those previously reported in the literature.[4]

2-(*sec*-butyl)-6-methylpyridine 1-oxide (3af)

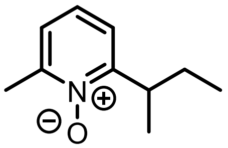

The reaction was conducted on a 0.2 mmol scale. The titled product was purified by column chromatography (hexane/acetone) to afford **3af** as a colorless oil (10.2 mg, 31% yield)

^{1}H NMR (400 MHz, $CDCl_3$) δ 7.19−6.98 (m, 3H), 3.70 (h, *J* = 7.0 Hz, 1H), 2.53 (s, 3H), 1.83−1.68 (m, 1H), 1.63−1.49 (m, 7.5 Hz, 1H), 1.24 (d, *J* = 7.0 Hz, 3H), 0.92 (t, *J* = 7.4 Hz, 3H).

^{13}C NMR (101 MHz, $CDCl_3$) δ 157.0, 149.2, 124.8, 123.6, 120.8, 34.3, 27.6, 18.7, 18.1, 11.9.

These spectroscopic data are consistent with those previously reported in the literature.[4]

2-methyl-6-(pentan-3-yl)pyridine 1-oxide (3ag)

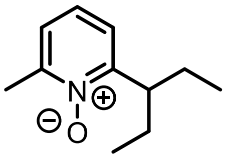

The reaction was conducted on a 0.2 mmol scale. The titled product was purified by column chromatography (hexane/acetone) to afford **3ag** as a colorless oil (6.8 mg, 19% yield)

^{1}H NMR (400 MHz, $CDCl_3$) δ 7.14–7.08 (m, 2H), 7.08–7.00 (m, 1H), 3.65 (p, *J* = 6.9 Hz, 1H),2.54 (s, 3H), 1.79–1.63 (m, 4H), 0.86 (t, *J* = 7.4 Hz, 6H).

^{13}C NMR (101 MHz, $CDCl_3$) δ 155.5, 149.4, 124.4, 123.5, 121.7, 41.1, 25.3, 18.8, 11.7.

ESI-HRMS (*m/z*): calculated for $C_{11}H_{17}NO$ ($[M+H]^+$): 180.1383, found: 180.1391.

2-isobutyl-6-methylpyridine 1-oxide (3ah)

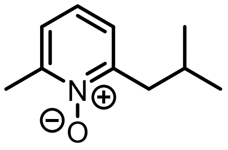

The reaction was conducted on a 0.2 mmol scale. The titled product was purified by column chromatography (hexane/acetone) to afford **3ah** as a colorless oil (5.0 mg, 15% yield)

^{1}H NMR (400 MHz, $CDCl_3$) δ 7.20–7.02 (m, 3H), 2.80 (d, *J* = 7.0 Hz, 2H), 2.53 (s, 3H), 2.35–2.21 (m, 1H), 0.97 (d, *J* = 6.7 Hz, 6H).

^{13}C NMR (101 MHz, $CDCl_3$) δ 151.7, 149.4, 124.5, 124.3, 124.0, 40.5, 25.4, 22.8, 18.6.

These spectroscopic data are consistent with those previously reported in the literature.[4]

2-(*tert*-butyl)-6-methylpyridine 1-oxide (3ai)

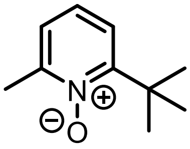

The reaction was conducted on a 0.2 mmol scale. The titled product was purified by column chromatography (hexane/acetone) to afford **3ai** as a colorless oil (5.6 mg, 17% yield)

^{1}H NMR (400 MHz, $CDCl_3$) δ 7.19–7.05 (m, 3H), 2.45 (s, 3H), 1.46 (s, 9H).

^{13}C NMR (101 MHz, $CDCl_3$) δ 157.9, 150.6, 124.5, 124.3, 121.4, 36.5, 27.4, 18.8

These spectroscopic data are consistent with those previously reported in the literature.[5]

2-cyclohexyl-6-phenylpyridine 1-oxide (3ba)

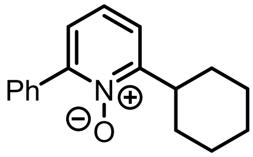

The reaction was conducted on a 0.2 mmol scale. The titled product was purified by column chromatography (hexane/acetone) to afford **3ba** as a colorless oil (29.9 mg, 59% yield)

^{1}H NMR (400 MHz, $CDCl_3$) δ 7.82–7.69 (m, 2H), 7.50–7.38 (m, 3H), 7.31–7.24 (m, 2H), 7.21 (m, *J* = 2.8 Hz, 1H), 3.58 (tt, *J* = 2.8 Hz, 1H), 2.13 (d, *J* = 11.6 Hz, 2H), 1.83 (m, 3H), 1.59–1.42 (m, 2H), 1.38–1.21 (m, 3H).

^{13}C NMR (101 MHz, $CDCl_3$) δ 157.4, 149.4, 133.8, 129.6, 129.2, 128.2, 125.1, 124.5, 121.8, 38.0, 31.1, 26.6, 26.5.

These spectroscopic data are consistent with those previously reported in the literature.[4]

2-cyclohexylquinoline 1-oxide (3ca)

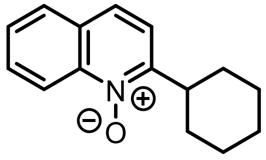

The reaction was conducted on a 0.2 mmol scale using 15 mol% of BINAP. The titled product was purified by column chromatography (hexane/acetone) to afford **3ca** as a colorless oil (31.8 mg, 70% yield).

44% yield (20.0 mg) under orange LED conditions; 24% yield (10.9 mg) under red LED conditions.

^{1}H NMR (400 MHz, $CDCl_3$) δ 8.80 (d, *J* = 8.8 Hz, 1H), 7.82 (d, *J* = 8.0 Hz, 1H), 7.78–7.68 (m, 2H), 7.64–7.53 (m, 1H), 7.33 (d, *J* = 8.7 Hz, 1H), 3.85 (t, *J* = 11.9 Hz, 1H), 2.16–2.06 (m,2H), 1.93–1.80 (m,3H), 1.64–1.51 (m,2H), 1.47–1.21 (m,3H)

^{13}C NMR (101 MHz, $CDCl_3$) δ 153.2, 141.8, 130.4, 128.8, 128.0, 127.8, 125.6, 120.1, 119.6, 38.0, 30.7, 26.5, 26.4.

These spectroscopic data are consistent with those previously reported in the literature.[6]

2-cyclohexyl-4-methylquinoline 1-oxide (3da)

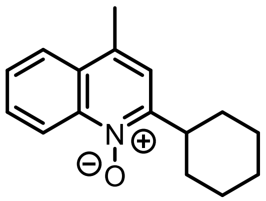

The reaction was conducted on a 0.2 mmol scale using 15 mol% of BINAP. The titled product was purified by column chromatography (hexane/acetone) to afford **3da** as a colorless oil (39.1 mg, 81% yield).

41% yield (19.8 mg) under orange LED conditions; 61% yield (29.4 mg) under red LED conditions; 71% NMR yield under blue LED conditions.

^{1}H NMR (400 MHz, $CDCl_3$) δ 8.85 (d, *J* = 8.7 Hz, 1H), 7.91 (d, *J* = 7.9 Hz, 1H), 7.81–7.68 (m, 1H), 7.66–7.54 (m, 1H), 7.14 (s, 1H), 3.84 (ddd, *J* = 12.0, 8.9, 3.1 Hz, 1H), 2.65 (s, 3H), 2.09 (d, *J* = 11.5 Hz, 2H), 1.93–1.79 (m, 3H), 1.65–1.50 (m, 2H), 1.44–1.23 (m, 3H).

^{13}C NMR (101 MHz, $CDCl_3$) δ 152.4, 141.2, 133.9, 130.1, 128.3, 127.6, 124.6, 120.7, 120.1, 37.9, 30.7, 26.5, 26.4, 18.6.

These spectroscopic data are consistent with those previously reported in the literature.[6]

2-cyclohexyl-6-methylquinoline 1-oxide (3ea)

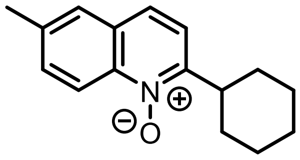

The reaction was conducted on a 0.2 mmol scale using 15 mol% of BINAP. The titled product was purified by column chromatography (hexane/acetone) to afford **3ea** as a colorless oil (29.9 mg, 62% yield)

^{1}H NMR (400 MHz, $CDCl_3$) δ 8.67 (d, *J* = 9.2 Hz, 1H), 7.66–7.52 (m, 3H), 7.29 (s, 1H), 3.82 (tt, *J* = 12.0, 3.2 Hz, 1H), 2.52 (s, 3H), 2.16–2.01 (m, 2H), 1.93–1.80 (m, 3H), 1.64–1.47 (tt, *J* = 12.7, 3.3 Hz, 2H), 1.44–1.30 (m, 3H).

^{13}C NMR (101 MHz, $CDCl_3$) δ 152.4, 140.4, 137.9, 132.6, 129.0, 126.9, 125.1, 120.0, 119.5, 38.0, 30.8, 26.6, 26.5, 21.5.

These spectroscopic data are consistent with those previously reported in the literature.[6]

2-cyclohexyl-6-methoxynaphthalene (3fa)

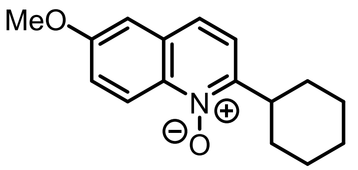

The reaction was conducted on a 0.2 mmol scale using 15 mol% of BINAP. Yield was determined by ^{1}H NMR analysis of the unpurified reaction mixture with dibromomethane as an internal standard (70% yield). Attempts to isolate **3fa** resulted in decomposition.

^{1}H NMR (400 MHz, $CDCl_3$) δ 8.69 (d, J = 9.6 Hz, 1H), 7.58 (d, J = 8.8 Hz, 1H), 7.36 (dd, J = 9.6, 2.8 Hz, 1H), 7.28 (s, 1H), 7.06 (d, J = 2.7 Hz, 1H), 3.92 (s, 3H), 3.83–3.73 (m, 1H), 2.15–2.05 (m, 2H), 1.91–1.77 (m, 3H), 1.62–1.48 (m, 2H), 1.41–1.25 (m, 3H).

^{13}C NMR (101 MHz, $CDCl_3$) δ 158.8, 151.2, 137.5, 130.1, 124.7, 122.5, 121.9, 120.1, 105.8, 55.8, 37.8, 30.8, 26.6, 26.4.

These spectroscopic data are consistent with those previously reported in the literature.[4]

2-cyclohexylbenzo[*h*]quinoline 1-oxide (3ga)

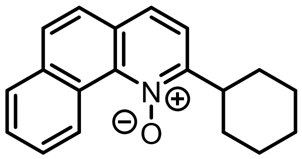

The reaction was conducted on a 0.2 mmol scale using 15 mol% of BINAP. The titled product was purified by column chromatography (hexane/acetone) to afford **3ga** as a colorless oil (41.0 mg, 74% yield).

70% yield (31.8 mg) under orange LED conditions; 64% yield (35.5 mg) under red LED conditions.

^{1}H NMR (400 MHz, $CDCl_3$) δ 10.99–10.92 (m, 1H), 7.97–7.89 (m, 1H), 7.83 (d, J = 8.7 Hz, 1H), 7.80—7.70 (m, 3H), 7.65 (d, J = 8.7 Hz, 1H), 7.47 (d, J = 8.3 Hz, 1H), 3.98–3.86 (m, 1H), 2.23–2.15 (m, 2H), 1.97–1.81 (m, 3H), 1.69–1.59 (m, 2H), 1.47–1.33 (m, 3H).

^{13}C NMR (101 MHz, $CDCl_3$) δ 156.2, 138.8, 134.7 129.9 129.3 128.8, 128.8 128.2, 127.6 126.8 125.3, 125.3, 119.6 38.5 31.2, 26.8 26.6.

These spectroscopic data are consistent with those previously reported in the literature.[4]

5. 8 mmol scale reaction

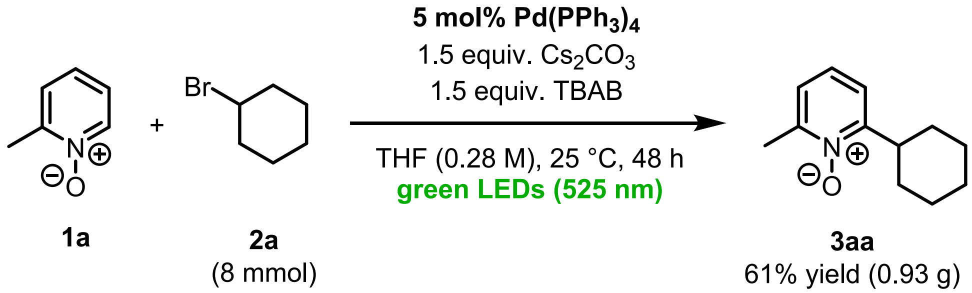

A flame-dried Schlenk tube equipped with a magnetic bar was charged with $Pd(PPh_3)_4$ (0.4 mmol, 5 mol%), Cs_2CO_3 (12.0 mmol, 1.5 equiv.) and TBAB (12.0 mmol, 1.5 equiv.). The tube was evacuated and backfilled with nitrogen; this process was repeated three times. **1a** (16.0 mmol, 2 equiv.) as a solution in THF (28 mL, 0.28 M) and alkyl bromide (8 mmol) were added. The Schlenk tube was capped and allowed to stir under green LED irradiation (40W, 525 nm) with fan cooling (reaction temperature: 25 °C; LEDs were placed directly against the Schlenk tube). After 48 h, the reaction mixture was transferred to a 100 mL round bottom flask and the solvent was removed

in vacuo. H_2O (50 mL) was added and the mixture was extracted with EtOAc (3 x 50 mL). The combined organic layers were washed with brine, dried over Na_2SO_4, filtered, and concentrated *in vacuo*. The residue was purified by column chromatography on silica gel (hexane/acetone) to afford the desired product (928.0 mg, 61% yield).

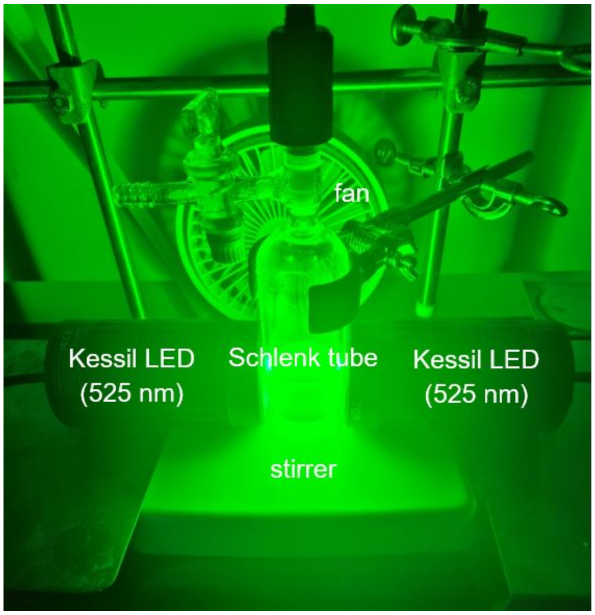

Figure S1. Experimental set-up for 8 mmol scale reaction.

6. Limitations

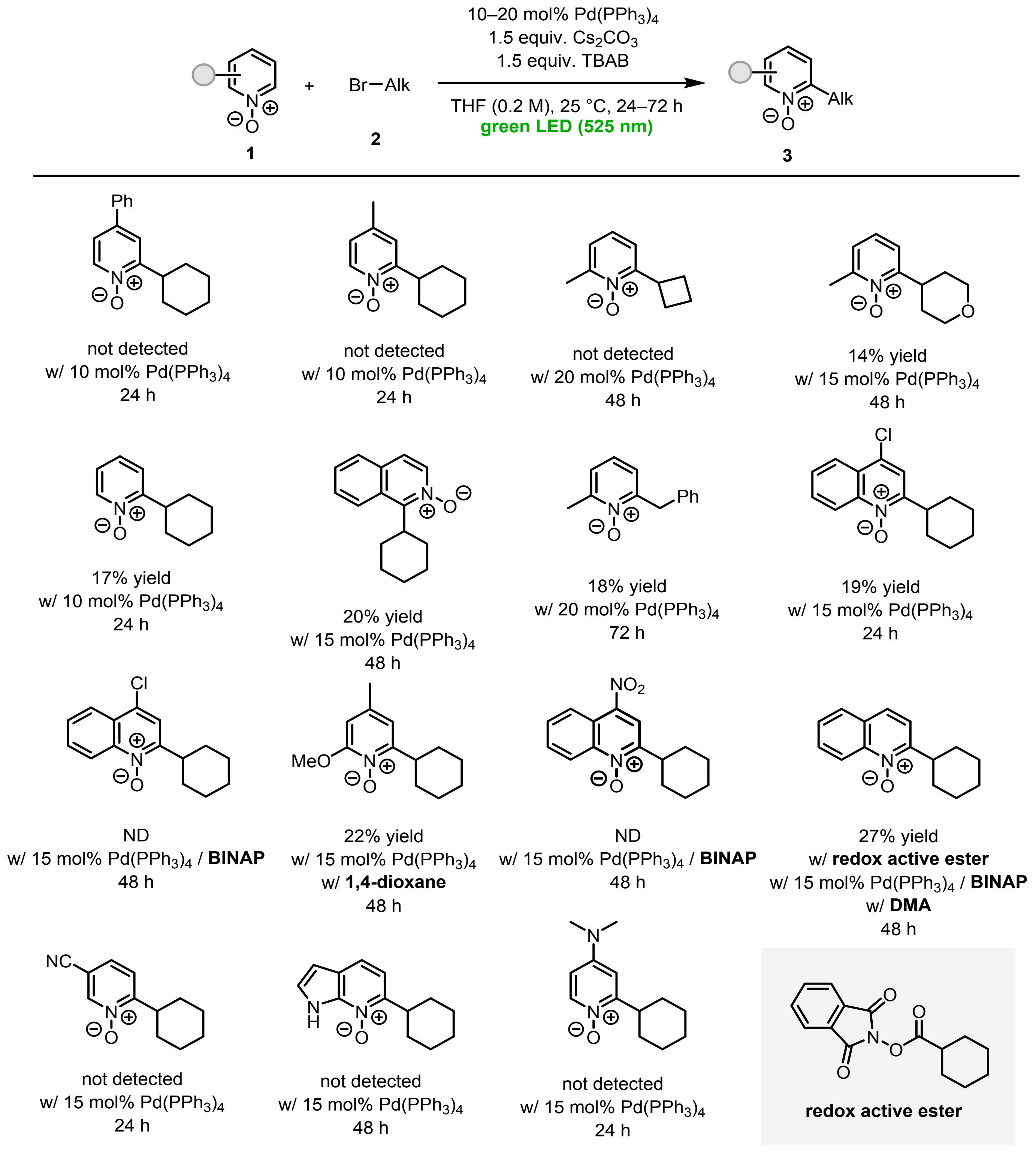

Scheme S1. Limitations.[a] [a]Reactions were conducted on 0.2 mmol scale. Yields were determined by ^{1}H NMR analysis with dibromomethane as an internal standard.

7. Radical trapping and control experiments

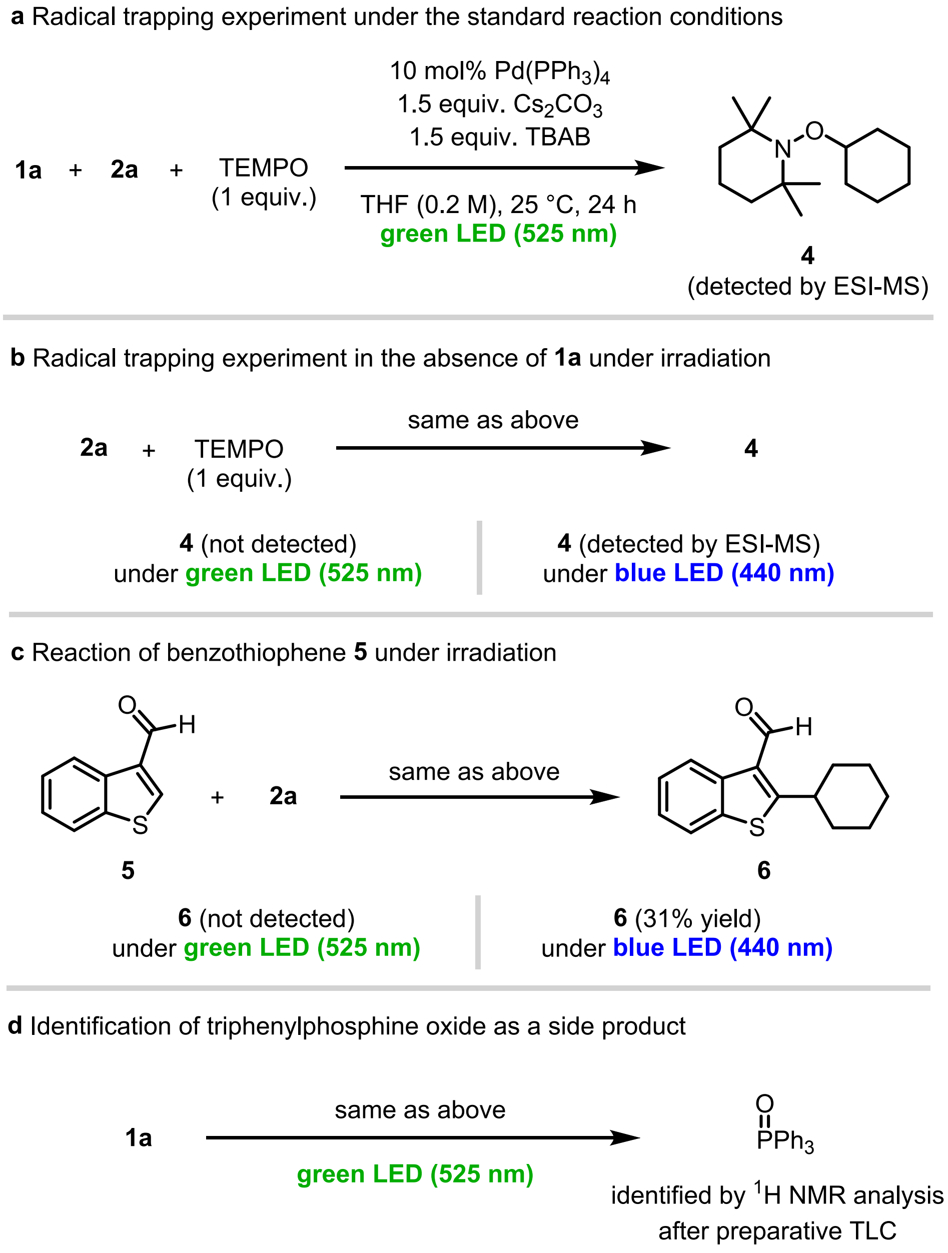

Scheme S2. Radical trapping and control experiments.[a] [a]Reactions were conducted on 0.2 mmol scale. Yields were determined by ^{1}H NMR analysis with dibromomethane as an internal standard.

Procedure for the experiment **a**: A flame-dried Schlenk tube equipped with a magnetic bar was charged with $Pd(PPh_3)_4$ (0.02 mmol, 10 mol%), Cs_2CO_3 (0.3 mmol, 1.5 equiv.), TBAB (0.3 mmol, 1.5 equiv.) and TEMPO (0.2 mmol, 1 equiv.). The tube was evacuated and backfilled with nitrogen; this process was repeated three times. **1a** (0.4 mmol, 2 equiv.) as a solution in THF (1 mL, 0.2 M) and **2a** (0.2 mmol) were added. The Schlenk tube was capped and allowed to stir under green LED irradiation (40W, 525 nm) with fan cooling (reaction temperature: 25 °C; distance between the LED and the Schlenk tube: 0.4 cm). After 24 h, the reaction mixture was transferred to a 100 mL round bottom flask and the solvent was removed *in vacuo*. H_2O (10 mL) was added and the mixture was extracted with

EtOAc (3 x 10 mL). The combined organic layers were washed with brine, dried over Na_2SO_4, filtered, and concentrated *in vacuo*. The mixture was then injected to electrospray ionization spectrometry (ESI-MS).

ESI-HRMS (*m/z*): calculated for $C_{15}H_{29}NO$ ($[M+H]^+$): 240.2327, found: 180.2326.

Procedure for the experiment **b**: A flame-dried Schlenk tube equipped with a magnetic bar was charged with $Pd(PPh_3)_4$ (0.02 mmol, 10 mol%), Cs_2CO_3 (0.3 mmol, 1.5 equiv.), TBAB (0.3 mmol, 1.5 equiv.) and TEMPO (0.2 mmol, 1 equiv.). The tube was evacuated and backfilled with nitrogen; this process was repeated three times. **2a** (0.2 mmol) and THF (1 mL, 0.2 M) were added. The Schlenk tube was capped and allowed to stir under either green LED (40W, 525 nm) or blue LED (40W, 440 nm) irradiation with fan cooling (reaction temperature: 25 °C; distance between the LED and the Schlenk tube: 0.4 cm). After 24 h, the reaction mixture was transferred to a 100 mL round bottom flask and the solvent was removed *in vacuo*. H_2O (10 mL) was added and the mixture was extracted with EtOAc (3 x 10 mL). The combined organic layers were washed with brine, dried over Na_2SO_4, filtered, and concentrated *in vacuo*. The mixture was then injected to electrospray ionization spectrometry (ESI-MS).

ESI-HRMS (*m/z*): calculated for $C_{15}H_{29}NO$ ($[M+H]^+$): 240.2327, found: 240.2325.

Procedure for the experiment **c**: A flame-dried Schlenk tube equipped with a magnetic bar was charged with $Pd(PPh_3)_4$ (0.02 mmol, 10 mol%), Cs_2CO_3 (0.3 mmol, 1.5 equiv.) and TBAB (0.3 mmol, 1.5 equiv.). The tube was evacuated and backfilled with nitrogen; this process was repeated three times. **5** (0.4 mmol, 2 equiv.) as a solution in THF (1 mL, 0.2 M) and **2a** (0.2 mmol) were added. The Schlenk tube was capped and allowed to stir under either green LED (40W, 525 nm) or blue LED (40W, 440 nm) irradiation (reaction temperature: 25 °C; distance between the LED and the Schlenk tube: 0.4 cm). After 24 h, the reaction mixture was transferred to a 100 mL round bottom flask and the solvent was removed *in vacuo*. H_2O (10 mL) was added and the mixture was extracted with EtOAc (3 x 10 mL). The combined organic layers were washed with brine, dried over Na_2SO_4, filtered, and concentrated *in vacuo*. Dibromomethane was added as an internal standard to determine the NMR yield.

Procedure for the experiment **d**: A flame-dried Schlenk tube equipped with a magnetic bar was charged with $Pd(PPh_3)_4$ (0.02 mmol, 10 mol%), Cs_2CO_3 (0.3 mmol, 1.5 equiv.) and TBAB (0.3 mmol, 1.5 equiv.). The tube was evacuated and backfilled with nitrogen; this process was repeated three times. **5** (0.4 mmol, 2 equiv.) as a solution in THF (1 mL, 0.2 M) was added. The Schlenk tube was capped and allowed to stir under green LED irradiation (40W, 525 nm) with fan cooling (reaction temperature: 25 °C; distance between the LED and the Schlenk tube: 0.4 cm). After 24 h, the reaction mixture was transferred to a 100 mL round bottom flask and the solvent was removed *in vacuo*. H_2O (10 mL) was added and the mixture was extracted with EtOAc (3 x 10 mL). The combined organic layers were washed with brine, dried over Na_2SO_4, filtered, and concentrated *in vacuo*. The residue was purified by preparative TLC on silica gel to afford the triphenylphosphine oxide.

8. UV-Vis-NIR absorption studies

UV-Vis-NIR absorption spectra were recorded on a Agilent 8453 UV-Vis Spectrophotometer, equipped with a temperature control unit at 25 C. The samples were measured in Starna Spectrosil® quartz cuvettes (path length = 1 mm) fitted with a PTFE stopper. The UV-Vis-NIR absorption of the reaction components $Pd(PPh_3)_4$, 2-methylpyridine *N*-oxide (**1a**), cyclohexyl bromide (**2a**), DIPEA, TBAB and their mixture were determined at the standard concentration (20 M) in THF as a solvent.

9. Titration experiments

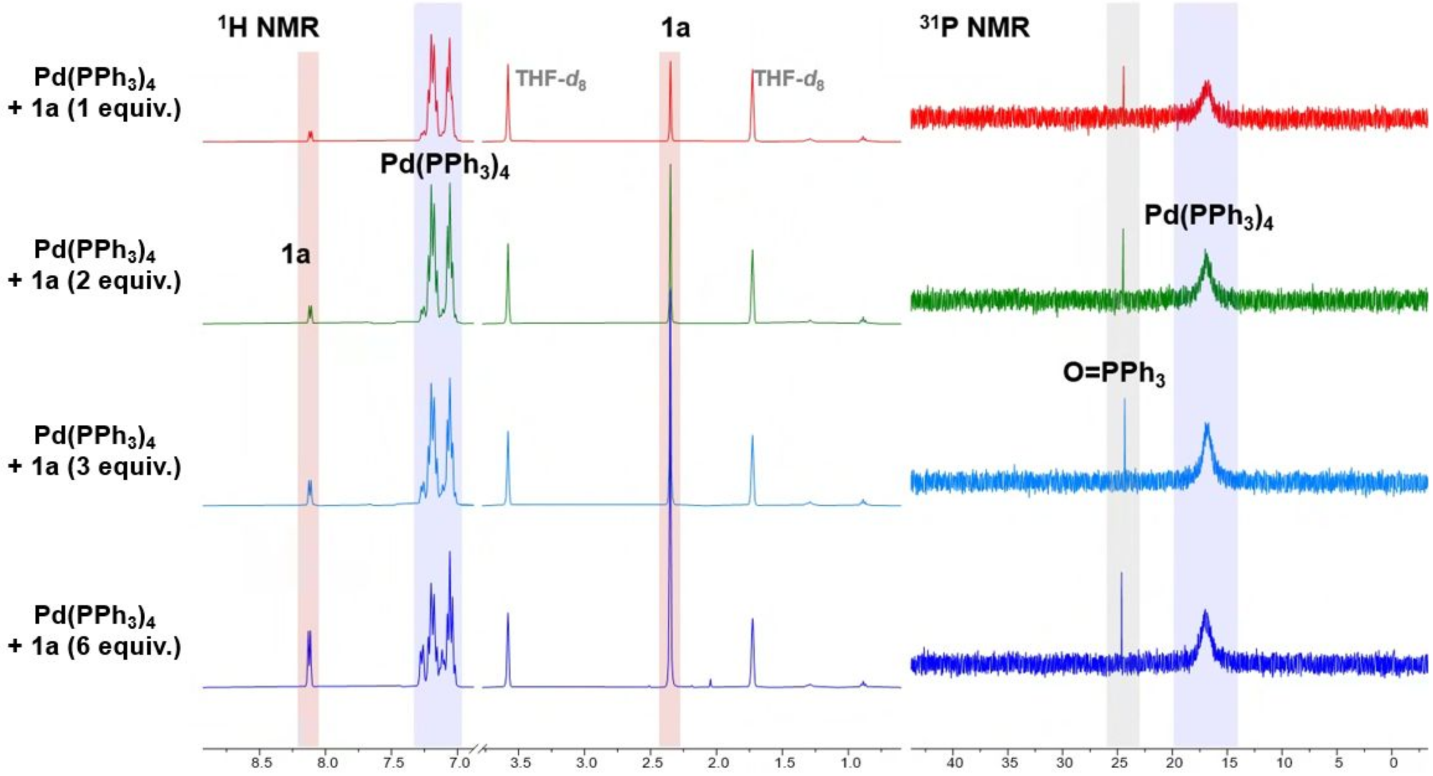

Figure S2. ^{1}H and ^{31}P NMR titration of $Pd(PPh_3)_4$ with **1a** in THF-d_8.

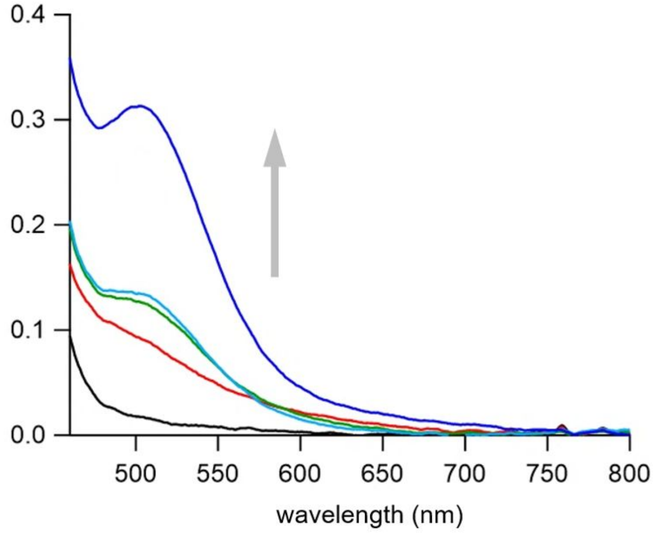

Figure S3. UV-Vis-NIR titration of $Pd(PPh_3)_4$ with **1a** in THF-d_8. $Pd(PPh_3)$:**1a** = 1:0 (black), 1:1 (red), 1:2 (green), 1:3 (light blue), and 1:6 (blue).

10. DFT calculations

10.1. Method

All DFT calculations were conducted by using the Gaussian 16-A.03 package,[7] with the B3LYP[8,9] functional in combination with the 6-311g(d,p)[10–12] basis set for main group atoms the LANL2TZ(f)[13–15] basis set including effective core potential for Pd atoms obtained from the Basis Set Exchange Software.[16] The CPCM solvation model was used with a dielectric constant of 7.43 for tetrahydrofuran.[17]

Table S4. DFT-optimized absolute energies (in unit of hartree/molecule) and the number of imaginary frequencies.

	$Pd(PPh_3)$·**1a**	$Pd(PPh_3)_4$	$Pd(PPh_3)_3$	$Pd(PPh_3)_2$	PPh_3	**1a**
**E**	-1526.175659	-4272.795586	-3236.318154	-2199.816276	-1036.497200	-362.872835
**H**	-1525.753421	-4271.627499	-3235.442711	-2199.232034	-1036.207803	-362.745189
**G**	-1525.844507	-4271.820249	-3235.595628	-2199.346476	-1036.271255	-362.783283
**Imaginary frequency**	0	0	0	0	0	0

Table S5. DFT-optimized absolute energies before and after 1a complexation. Entropy corrections were applied based on optimizations in which all reactants and products were treated collectively in a single simulation box.

	$Pd(PPh_3)_4$ + **1a** (Reactant, R)	$Pd(PPh_3)$·**1a** + 3 PPh_3 (Product, P)	P-R (kcal/mol)
**E**	–4635.668926	–4635.670538	–1.01
**H**	–4634.371108	–4634.373901	–1.75
**G**	–4634.590400	–4634.616798	–16.56

10.2. Cartesian coordinates

Pd(PPh$_3$)·1a complex

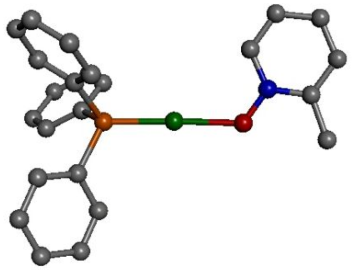

Pd	0.89959	-0.947861	-0.527883	H	6.384992	-2.719439	-3.983224
O	-1.223035	-0.515802	-0.681111	H	2.675714	-4.883256	-3.962143
N	-1.845755	0.380198	0.050333	H	4.918303	-4.472637	-4.949676
C	-1.171157	1.114986	0.976767	C	4.208513	0.014377	-0.33626
C	-3.191409	0.552796	-0.146325	C	5.366958	0.036234	0.449238
C	-1.822326	2.055836	1.746818	C	3.883179	1.152494	-1.088557
H	-0.113745	0.882339	1.034174	C	6.183229	1.167792	0.476769
C	-3.858415	1.49775	0.623733	H	5.635726	-0.827091	1.045413
C	-3.837986	-0.300114	-1.184828	C	4.70472	2.276328	-1.071052
C	-3.189512	2.258293	1.57546	H	2.973019	1.15496	-1.679454
H	-1.250892	2.619305	2.472411	C	5.857331	2.28785	-0.284713
H	-4.92068	1.624948	0.459702	H	7.074285	1.170965	1.094836
H	-3.369498	-0.13942	-2.159142	H	4.439735	3.146252	-1.661687
H	-3.709017	-1.358898	-0.946043	H	6.492434	3.166246	-0.261519
H	-4.900549	-0.069366	-1.249953	C	3.596946	-2.487388	1.001892
H	-3.72117	2.990824	2.168625	C	2.930913	-2.313438	2.223093
P	3.056804	-1.44036	-0.435286	C	4.625554	-3.434541	0.925047
C	3.718498	-2.385277	-1.891067	C	3.295927	-3.053818	3.344846
C	4.979787	-2.161014	-2.4557	H	2.114617	-1.600916	2.282523
C	2.893374	-3.369725	-2.453665	C	4.983421	-4.18418	2.045778
C	5.407421	-2.908673	-3.553666	H	5.148658	-3.593887	-0.010126
H	5.631903	-1.400204	-2.044214	C	4.322943	-3.993736	3.258014
C	3.325539	-4.124186	-3.541066	H	2.771651	-2.905177	4.282309
H	1.9034	-3.53165	-2.039806	H	5.778784	-4.917315	1.969253
C	4.584624	-3.893287	-4.096181	H	4.601606	-4.578153	4.127667

Pd(PPh$_3$)$_4$ complex

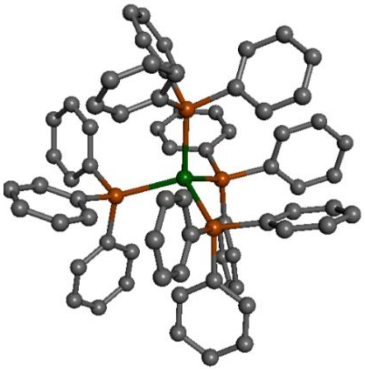

Pd	4.003526	3.997951	3.998496	C	7.913848	2.91334	0.996122
P	5.47768	5.471229	5.474278	C	3.619696	0.48678	1.915517
P	2.507374	5.473533	2.515065	C	4.944573	1.136986	0.01613
C	6.68753	6.620579	4.633966	C	4.344071	7.803535	8.759258

C	6.870623	7.960669	4.999959	H	5.802528	6.279085	8.394742
C	7.810368	8.757149	4.344158	C	2.951012	8.414275	6.893971
C	8.593396	8.225957	3.321835	H	3.278269	7.330765	5.079692
C	8.423639	6.892326	2.95163	C	8.761823	4.336336	7.807161
C	7.47298	6.103694	3.594819	H	8.398604	5.793737	6.281391
C	3.339134	6.704046	1.382436	C	6.895013	2.946222	8.41987
C	3.003781	6.8755	0.033132	H	5.080732	3.274634	7.336787
C	3.652439	7.834817	-0.746126	C	-0.746519	3.643266	7.826797
C	4.636602	8.647637	-0.188519	H	-0.4157	2.224792	6.256516
C	4.97644	8.488842	1.154843	C	1.153208	4.96661	8.485646
C	4.340445	7.520075	1.926523	H	2.96183	4.624458	7.397442
C	1.277388	6.605648	3.350111	C	5.090288	-0.408162	6.880876
C	0.565618	6.109161	4.44912	H	4.832187	0.777556	5.118085
C	-0.405641	6.879974	5.084313	C	3.558705	0.034083	8.685023
C	-0.668274	8.176075	4.643294	H	2.063724	1.522114	8.317073
C	0.043582	8.689108	3.560686	C	-0.460053	1.142187	2.952587
C	1.003419	7.909571	2.914768	H	0.604108	2.957318	3.329746
C	1.379259	4.621556	1.294653	C	0.198473	-0.765673	4.263708
C	1.924641	3.614809	0.486161	H	1.745448	-0.43652	5.707164
C	1.149674	2.95967	-0.467331	C	7.83717	-0.748007	3.643887
C	-0.198321	3.2843	-0.617122	H	6.26842	-0.418037	2.223471
C	-0.757475	4.2729	0.189113	C	8.491927	1.151569	4.969511
C	0.024847	4.941109	1.132299	H	7.402087	2.959138	4.627018
H	6.284356	8.391773	5.800728	C	6.888363	5.088828	-0.406763
H	7.932675	9.793023	4.641281	H	5.124581	4.82803	0.776868
H	9.326082	8.844878	2.816316	C	8.694208	3.559775	0.03733
H	9.020615	6.465895	2.153674	H	8.32676	2.064147	1.524851
H	7.3407	5.077489	3.277307	C	2.964324	-0.465368	1.139113
H	2.232147	6.265531	-0.418097	H	3.339348	0.598316	2.954944
H	5.136216	9.39519	-0.794315	C	4.276121	0.195127	-0.767597
H	5.744896	9.108844	1.602228	H	5.717979	1.743233	-0.436855
H	4.632839	7.397885	2.961556	C	3.320858	8.585079	8.227565
H	0.777222	5.115939	4.822323	H	4.640896	7.926674	9.795118
H	-0.944739	6.469619	5.930486	H	2.152354	9.010057	6.46718
H	-1.416914	8.781972	5.141262	C	8.22913	3.314581	8.589895
H	-0.150496	9.696967	3.210216	H	9.798067	4.632066	7.9297
H	1.534577	8.324149	2.067911	H	6.46742	2.148753	9.016666
H	2.963636	3.333795	0.599944	C	-0.190406	4.626505	8.641741
H	1.598984	2.187978	-1.081882	H	1.599466	5.734343	9.107351
H	-0.806518	2.76934	-1.352213	C	4.644968	-0.674242	8.174784
H	-1.804745	4.534113	0.082891	H	5.939314	-0.944477	6.472771
H	-0.428762	5.715007	1.737459	H	3.204883	-0.162699	9.691185
P	2.518987	2.500962	5.469974	C	-0.608017	-0.206457	3.27536
P	5.478014	2.515476	2.500764	H	-1.074839	1.591562	2.181118
C	4.635502	6.680129	6.623232	H	0.093702	-1.813445	4.52349
C	6.625826	4.629229	6.684042	C	8.650053	-0.191625	4.628687
C	1.384025	3.331412	6.699314	H	9.111968	1.59804	5.738466
C	3.353127	1.271196	6.603025	C	8.183205	4.645386	-0.671441
C	1.300529	1.372307	4.615902	H	6.479633	5.937322	-0.943444
C	6.707651	1.381419	3.33193	H	9.701085	3.207403	-0.158391
C	6.611158	3.351117	1.272136	C	3.288119	-0.612473	-0.209388
C	4.625897	1.29663	1.371065	H	2.193114	-1.081023	1.587737
C	5.001121	6.864233	7.96329	H	4.536713	0.091023	-1.815234
C	3.595484	7.464052	6.105853	H	2.814369	9.317424	8.846098

C	7.966363	4.993398	6.867414	H	8.847243	2.808085	9.322588
C	6.107388	3.590649	7.469229	H	-0.797515	5.125184	9.388852
C	0.034456	2.995775	6.868082	H	5.142428	-1.422794	8.78121
C	1.926606	4.331823	7.517467	H	-1.341889	-0.815075	2.759163
C	4.455704	0.563023	6.10943	H	9.397134	-0.798232	5.128023
C	2.913472	0.993786	7.90479	H	8.789752	5.143791	-1.419265
C	0.491842	1.917762	3.60933	H	2.772972	-1.346539	-0.818662
C	1.140055	0.017217	4.933524	H	-1.790099	3.369922	7.93855
C	6.878441	0.032281	2.995592	H	7.95054	-1.791267	3.369991
C	7.523767	1.924269	4.333846	H	3.379331	7.948692	-1.789537
C	6.116749	4.453041	0.563495				

Pd(PPh$_3$)$_3$ complex

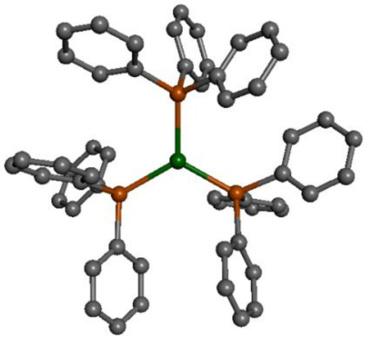

Pd	3.247378	3.242457	3.2407	C	3.651422	0.13339	1.378613
P	2.185499	5.195977	2.328641	C	5.661918	0.414356	0.080042
C	3.361256	6.498482	1.7191	C	0.140433	4.116449	8.172096
C	3.193404	7.214701	0.528371	H	-0.103205	2.32739	7.016917
C	4.126066	8.177654	0.141558	C	2.130475	5.403576	7.736063
C	5.233509	8.445802	0.943235	H	3.424706	4.640023	6.201195
C	5.410918	7.738008	2.132294	C	4.725201	-1.003692	6.269536
C	4.487891	6.766054	2.510088	H	3.823857	-0.210863	4.496545
C	1.003277	6.222026	3.340769	C	4.343451	0.233659	8.299486
C	-0.080122	5.567606	3.946345	H	3.100227	1.971587	8.128918
C	-0.999097	6.270734	4.718834	C	-0.990129	0.631592	3.308627
C	-0.840832	7.64272	4.92199	H	0.481919	2.167154	2.977644
C	0.239367	8.300707	4.340824	C	-0.708546	-0.671426	5.317077
C	1.152217	7.597847	3.55169	H	0.958652	-0.149683	6.559821
C	1.132514	4.826511	0.841629	C	8.177586	0.135375	4.117976
C	1.389061	3.647924	0.128836	H	7.023871	-0.107233	2.327811
C	0.639307	3.316811	-0.998993	C	7.741326	2.125286	5.405228
C	-0.387884	4.15667	-1.425159	H	6.207735	3.420428	4.64069
C	-0.66248	5.325907	-0.716306	C	6.280581	4.726681	-0.999563
C	0.090254	5.658401	0.408787	H	4.506618	3.825415	-0.208765
H	2.336722	7.023651	-0.10572	C	8.309846	4.34113	0.237748
H	5.956569	9.195288	0.641917	H	8.137714	3.095042	1.973473
H	6.273441	7.934166	2.759441	C	3.319439	-0.992848	0.62687
H	4.645325	6.201406	3.423312	H	2.986843	0.477711	2.16352
H	-0.206245	4.498651	3.815743	C	5.328543	-0.709151	-0.674687
H	-1.833252	5.746043	5.17109	H	6.570245	0.958126	-0.150675
H	-1.551456	8.189979	5.53098	C	0.940512	5.224624	8.441948
H	0.373964	9.366036	4.493364	H	2.756421	6.266666	7.93356
H	1.978272	8.131122	3.098833	C	4.92639	-0.8458	7.641854
H	2.173204	2.982955	0.47413	H	5.178825	-1.837282	5.745129
H	0.852567	2.399626	-1.536559	H	4.49442	0.367933	9.365081

H	-0.976067	3.898906	-2.298712
H	-1.464723	5.980749	-1.038045
H	-0.141374	6.566339	0.952818
P	2.333933	2.179452	5.192806
P	5.200929	2.330627	2.178961
C	1.721368	3.354009	6.495023
C	3.345616	0.997607	6.219756
C	0.848342	1.125338	4.820772
C	6.50188	1.717262	3.354489
C	6.229058	3.343706	0.999343
C	4.830448	0.845874	1.123175
C	0.52971	3.184633	7.209322
C	2.510753	4.481364	6.764305
C	3.95294	-0.085049	5.565722
C	3.554535	1.146151	7.595923
C	0.136626	1.382138	3.641587
C	0.415486	0.082089	5.651403
C	7.215795	0.525334	3.185421
C	6.770546	2.506245	4.482278
C	5.575862	3.953394	-0.082468
C	7.605363	3.551212	1.148652
C	-1.416306	-0.396625	4.14723
H	-1.526863	0.845063	2.391002
H	-1.030307	-1.474425	5.970979
C	8.446836	0.935044	5.226586
H	7.938365	2.750924	6.268646
C	7.653003	4.926496	-0.840888
H	5.756786	5.1822	-1.832504
H	9.37552	4.491008	0.372605
C	4.158866	-1.417706	-0.401223
H	2.401933	-1.5302	0.839294
H	5.983066	-1.029885	-1.477592
H	0.637284	5.947052	9.191273
H	5.535203	-1.556154	8.189658
H	-2.289028	-0.985409	3.888028
H	9.195401	0.631302	5.949586
H	8.201518	5.536127	-1.549992
H	3.900431	-2.290017	-0.990952
H	-0.789233	3.97281	8.711729
H	8.716931	-0.794491	3.974555
H	3.983657	8.718789	-0.787423

$Pd(PPh_3)_2$ complex

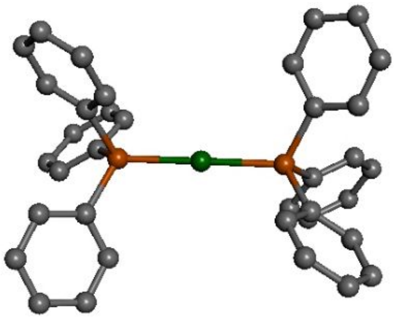

Pd	3.986538	3.993089	1.645672
P	2.329489	5.621339	1.645174
C	2.87993	7.295369	1.075942
C	2.088768	8.145403	0.294142
C	2.570258	9.394057	-0.09985
C	3.842837	9.809424	0.286488
C	4.639333	8.967993	1.063629
C	4.164773	7.71666	1.447695
C	1.562607	5.96152	3.29584
C	1.390003	4.87609	4.166493
C	0.809062	5.056536	5.419097
C	0.407838	6.328848	5.826602
C	0.587313	7.416229	4.973966
C	1.159115	7.235192	3.714516
C	0.874207	5.253837	0.56132
C	1.119588	4.592284	-0.650316
C	0.073386	4.289691	-1.518006
C	-1.236384	4.629388	-1.178677
C	-1.491923	5.276309	0.028693
C	-0.443506	5.590007	0.893817
H	1.097131	7.835963	-0.013116
C	6.411867	2.013755	3.279948
C	7.097283	2.738535	0.54924
C	5.091682	0.693981	1.053599
C	6.814979	0.737293	3.690373
C	6.586363	3.093781	4.156932
C	6.851104	3.410351	-0.656556
C	8.414972	2.397531	0.876946
C	3.809168	0.268539	1.428682
C	5.879757	-0.149415	0.2616
C	7.388167	0.548212	4.94801
H	6.6797	-0.113631	3.033723
C	7.168719	2.905328	5.407697
H	6.252342	4.081759	3.857344
C	7.89645	3.718225	-1.523432
H	5.835778	3.701242	-0.905415
C	9.462543	2.716446	0.012715
H	8.629649	1.887478	1.808213
C	3.334015	-0.980458	1.037852
H	3.181094	0.927242	2.019551
C	5.397589	-1.395666	-0.139177
H	6.869527	0.163296	-0.048292

H	4.21545	10.779886	-0.021249	C	7.569481	1.630269	5.807008
H	5.633917	9.281067	1.360813	H	7.298682	3.750798	6.073847
H	4.795077	7.052776	2.030347	C	9.206162	3.373558	-1.188966
H	1.724335	3.890204	3.860477	H	7.690067	4.236743	-2.452965
H	0.680541	4.206963	6.080288	H	10.478867	2.45051	0.281147
H	-0.035405	6.472288	6.805518	C	4.127455	-1.815292	0.250519
H	0.283104	8.408773	5.287047	H	2.341295	-1.296808	1.337788
H	1.292997	8.090201	3.062909	H	6.016965	-2.037248	-0.755965
H	2.135005	4.30526	-0.903299	H	8.013821	1.48056	6.784487
H	0.279188	3.779158	-2.452076	H	10.022057	3.621695	-1.858323
H	-2.052937	4.385321	-1.848725	H	3.754335	-2.783893	-0.062422
H	-2.508261	5.538347	0.300889	H	7.692056	-0.44643	5.254687
H	-0.657513	6.092183	1.829506	H	1.948502	10.040843	-0.708758
P	5.643144	2.364413	1.632397				

11. References

1. Roudesly, F.; Veiros, L. F.; Oble, J.; Poli, G. *Org. Lett.* **2018**, *20*, 2346–2350.

2. Goldschmid, S. L.; Tay, N. E. S.; Joe, C. L.; Lainhart, B. C.; Sherwood, T. C.; Simmons, E. M.; Sezen-Edmonds, M.; Rovis, T. *J. Am. Chem. Soc.* **2022**, *144*, 22409–22415.

3. Xie, K. A.; Bednarova, E.; Joe, C. L.; Sherwood, T. C.; Welin, E. R.; Rovis, T. *J. Am. Chem. Soc.* **2024**, *146*, 25780–25787.

4. Xiao, B.; Liu, Z.-J.; Liu, L.; Fu, Y. *J. Am. Chem. Soc.* **2013**, *135*, 616–619.

5. Cai, C.-Y.; Chen, S.-J.; Merchant, R. R.; Kanda, Y.; Qin, T. *J. Am. Chem. Soc.* **2024**, *146*, 24257–24264.

6. Qin, P.-T.; Sun, J.; Wang, F.; Wang, J.-Y.; Wang, H.; Zhou, M.-D. *Adv. Synth. Catal.* **2020**, *362*, 4707–4715.

7. Frisch, M. J.; Trucks, G. W.; Schlegel, H. B.; Scuseria, G. E.; Robb, M. A.; Cheeseman, J. R.; Scalmani, G.; Barone, V.; Petersson, G. A.; Nakatsuji, H.; Li, X.; Caricato, M.; Marenich, A. V.; Bloino, J.; Janesko, B. G.; Gomperts, R.; Mennucci, B.; Hratchian, H. P.; Ortiz, J. V.; Izmaylov, A. F.; Sonnenberg, J. L.; Williams-Young, D.; Ding, F.; Lipparini, F.; Egidi, F.; Goings, J.; Peng, B.; Petrone, A.; Henderson, T.; Ranasinghe, D.; Zakrzewski, V. G.; Gao, J.; Rega, N.; Zheng, G.; Liang, W.; Hada, M.; Ehara, M.; Toyota, K.; Fukuda, R.; Hasegawa, J.; Ishida, M.; Nakajima, T.; Honda, Y.; Kitao, O.; Nakai, H.; Vreven, T.; Throssell, K.; Montgomery, J. A., Jr.; Peralta, J. E.; Ogliaro, F.; Bearpark, M. J.; Heyd, J. J.; Brothers, E. N.; Kudin, K. N.; Staroverov, V. N.; Keith, T. A.; Kobayashi, R.; Normand, J.; Raghavachari, K.; Rendell, A. P.; Burant, J. C.; Iyengar, S. S.; Tomasi, J.; Cossi, M.; Millam, J. M.; Klene, M.; Adamo, C.; Cammi, R.; Ochterski, J. W.; Martin, R. L.; Morokuma, K.; Farkas, O.; Foresman, J. B.; Fox, D. J. *Gaussian 16 Rev. A.03*, Wallingford, CT, **2016**.

8. Becke, A. D. *Phys. Rev. A* **1988**, *38*, 3098–3100.

9. Lee, C.; Yang, W.; Parr, R. G. *Phys. Rev. B* **1988**, *37*, 785–789.

10. Krishnan, R.; Binkley, J. S.; Seeger, R.; Pople, J. A. *J. Chem. Phys.* **1980**, *72*, 650–654.

11. McLean, A. D.; Chandler, G. S. *J. Chem. Phys.* **1980**, *72*, 5639–5648.

12. Francl, M. M.; Pietro, W. J.; Hehre, W. J.; Binkley, J. S.; Gordon, M. S.; DeFrees, D. J.; Pople, J. A. *J. Chem. Phys.* **1982**, *77*, 3654–3665.

13. Hay, P. J.; Wadt, W. R. *J. Chem. Phys.* **1985**, *82*, 299–310.

14. Ehlers, A. W.; Böhme, M.; Dapprich, S.; Gobbi, A.; Höllwarth, A.; Jonas, V.; Köhler, K. F.; Stegmann, R.; Veldkamp, A.; Frenking, G. *Chem. Phys. Lett.* **1993**, *208*, 111–114.

15. Roy, L. E.; Hay, P. J.; Martin, R. L. *J. Chem. Theory Comput.* **2008**, *4*, 1029–1031.

16. Pritchard, B. P.; Altarawy, D.; Didier, B.; Gibson, T. D.; Windus, T. L. *J. Chem. Inf. Model.* **2019**, *59*, 4814–4820.

17. Barone, V.; Cossi, M. J. *Phys. Chem. A* **1998**, *102*, 1995–2001.

12. Copies of NMR spectra

2-cyclohexyl-6-methylpyridine 1-oxide (3aa)

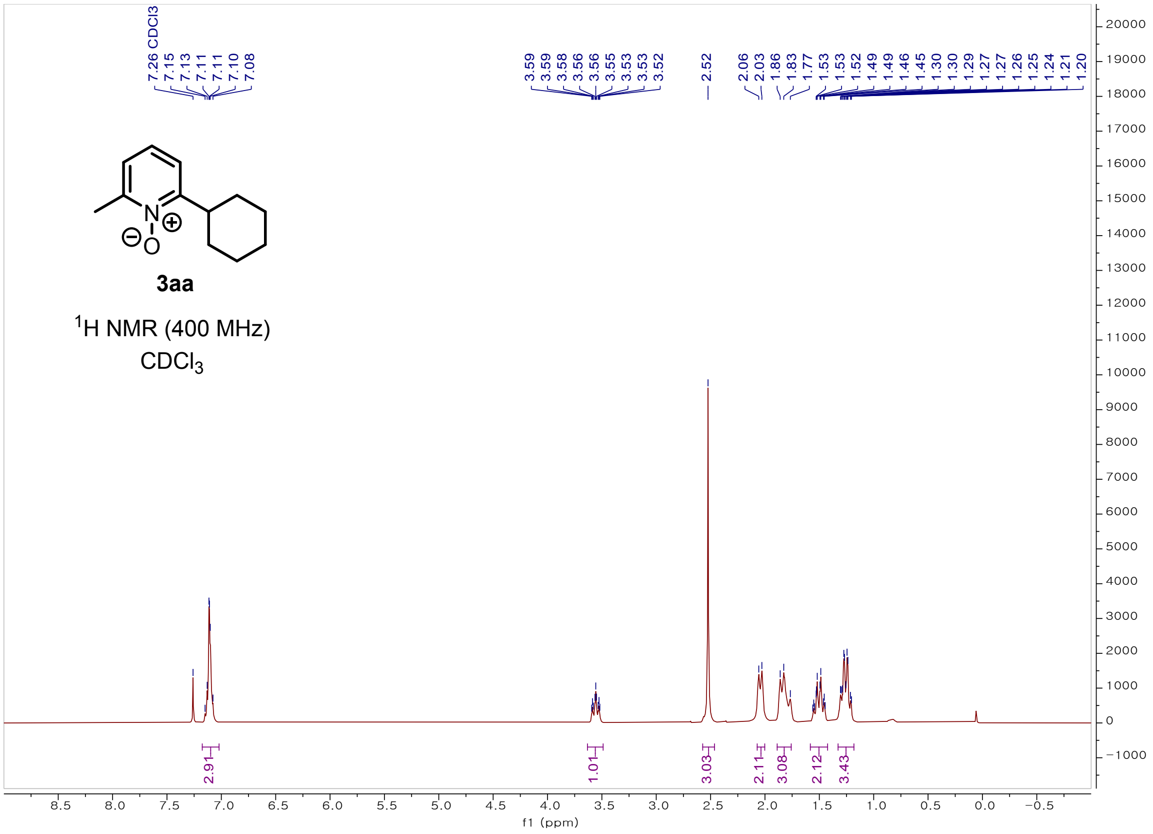

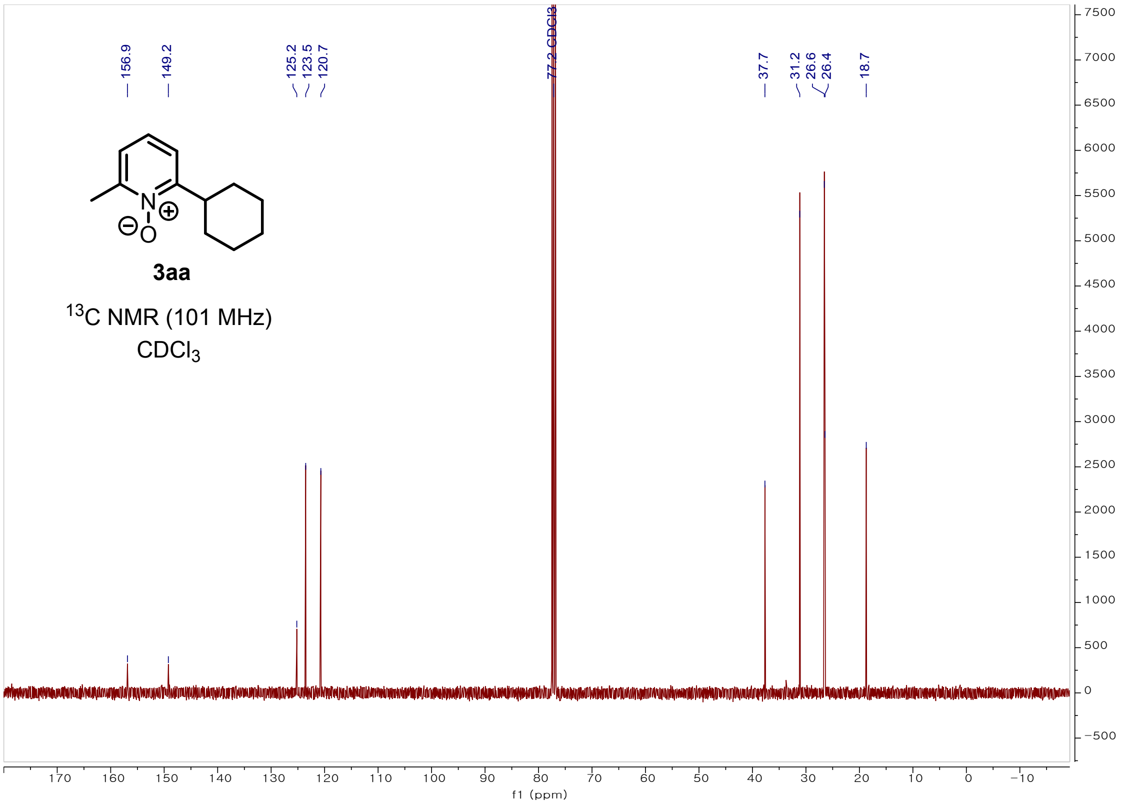

2-cyclopentyl-6-methylpyridine 1-oxide (3ab)

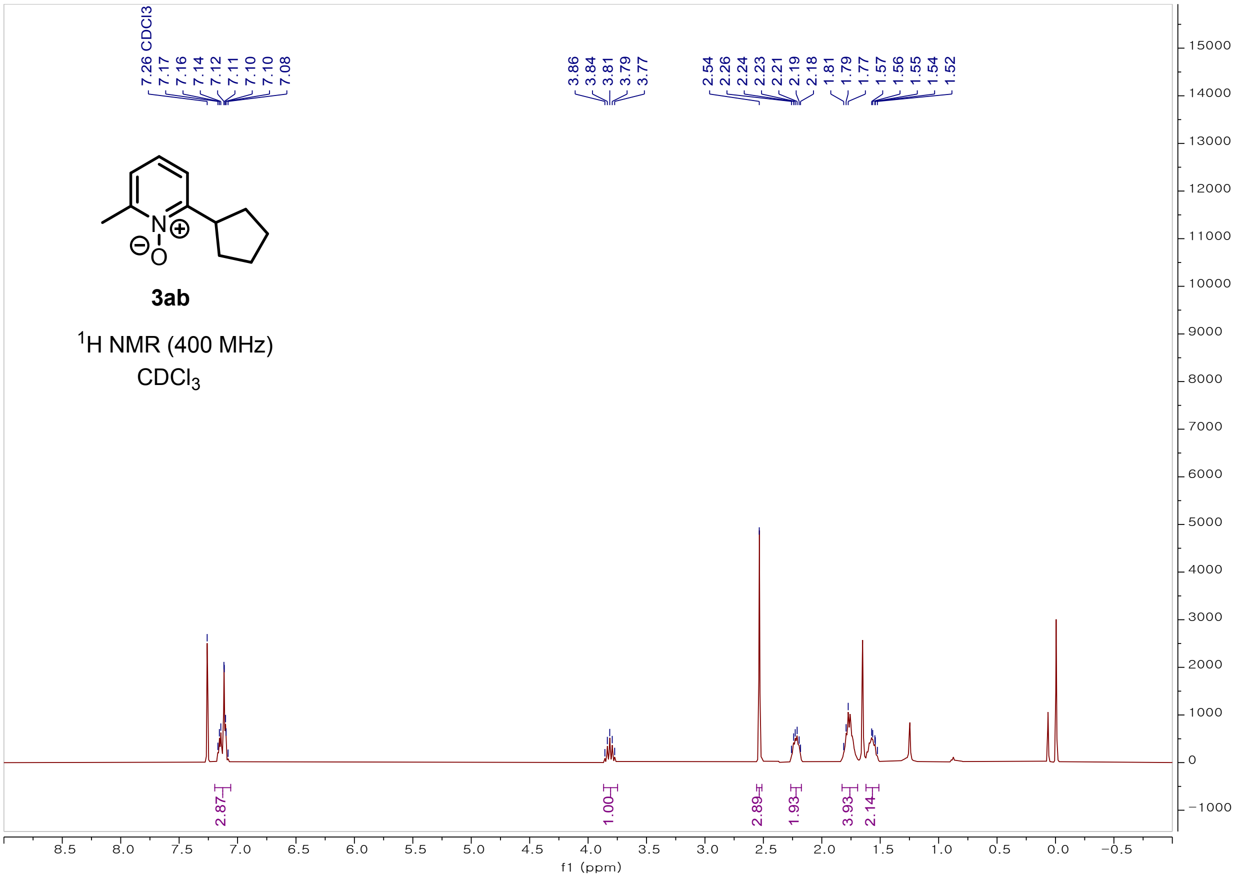

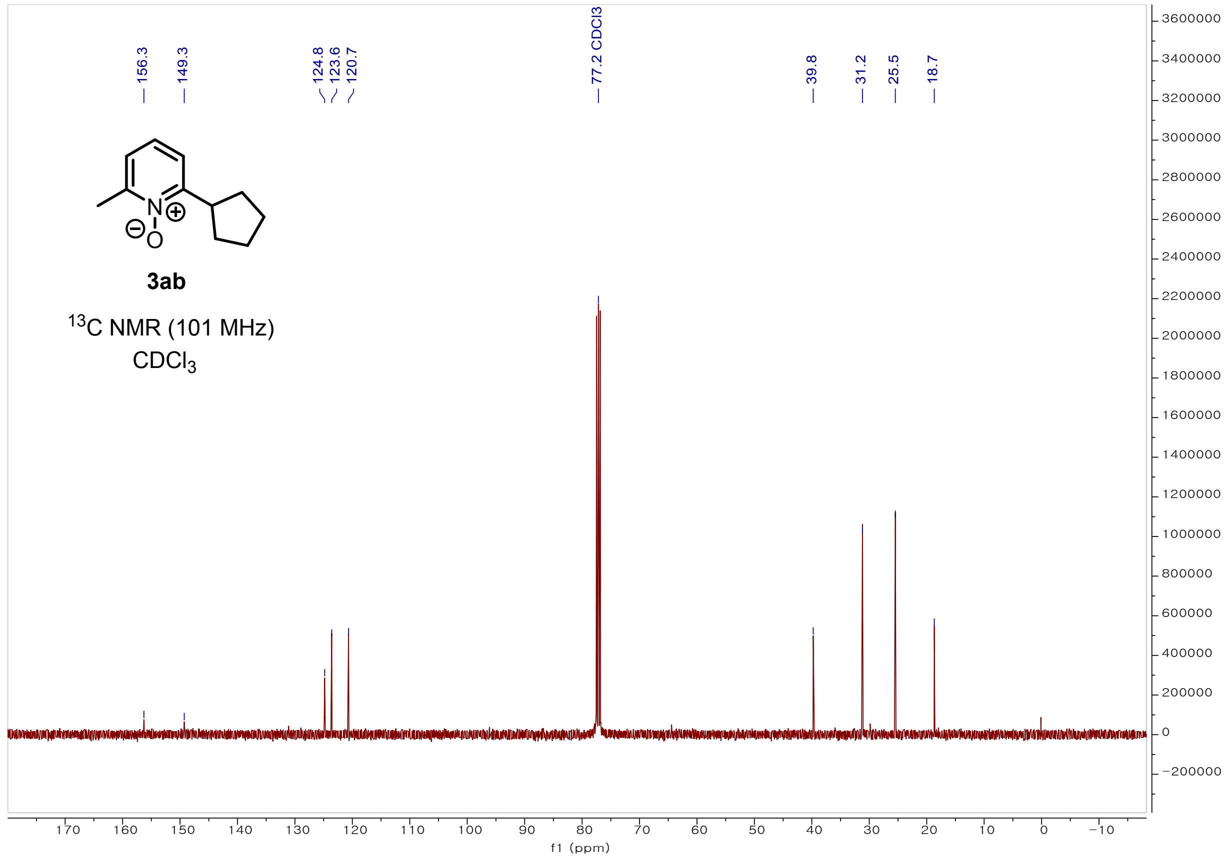

2-cycloheptyl-6-methylpyridine 1-oxide (3ac)

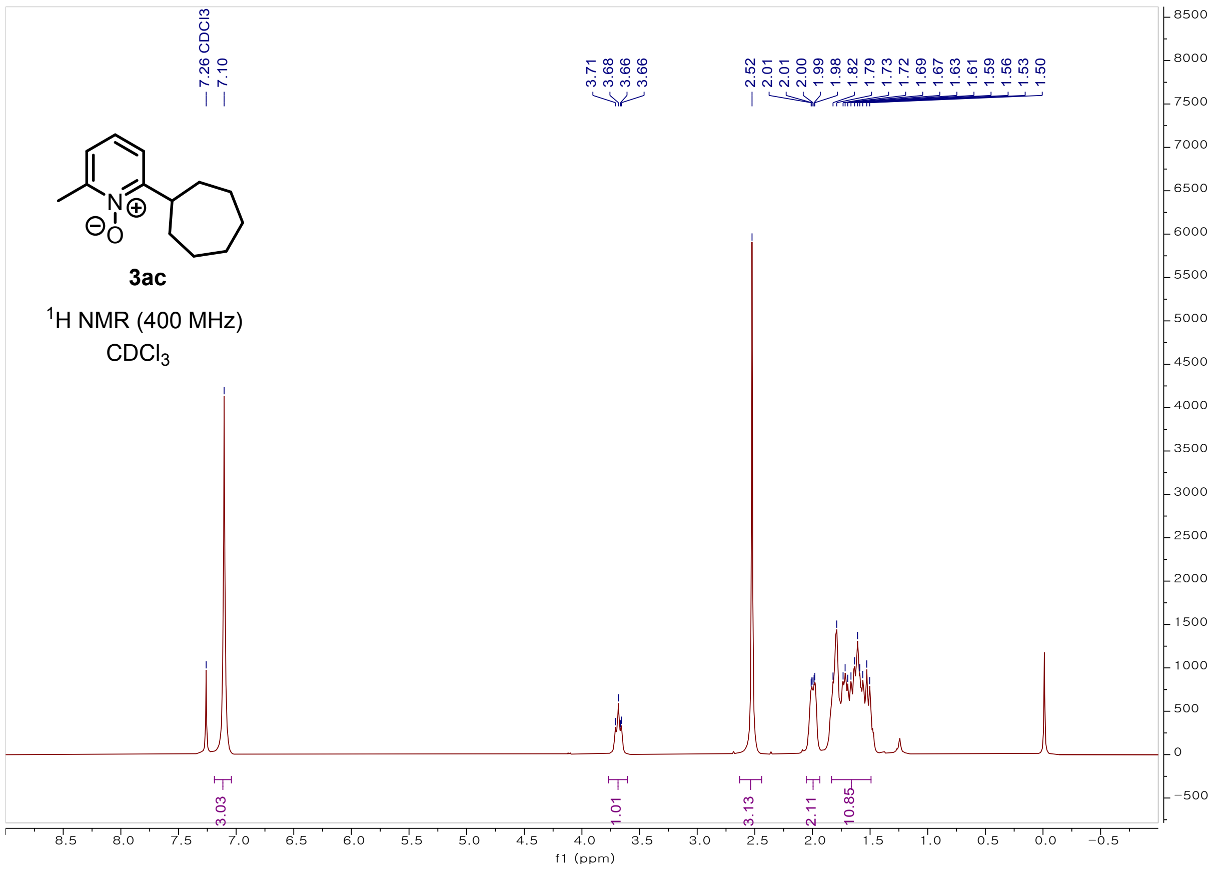

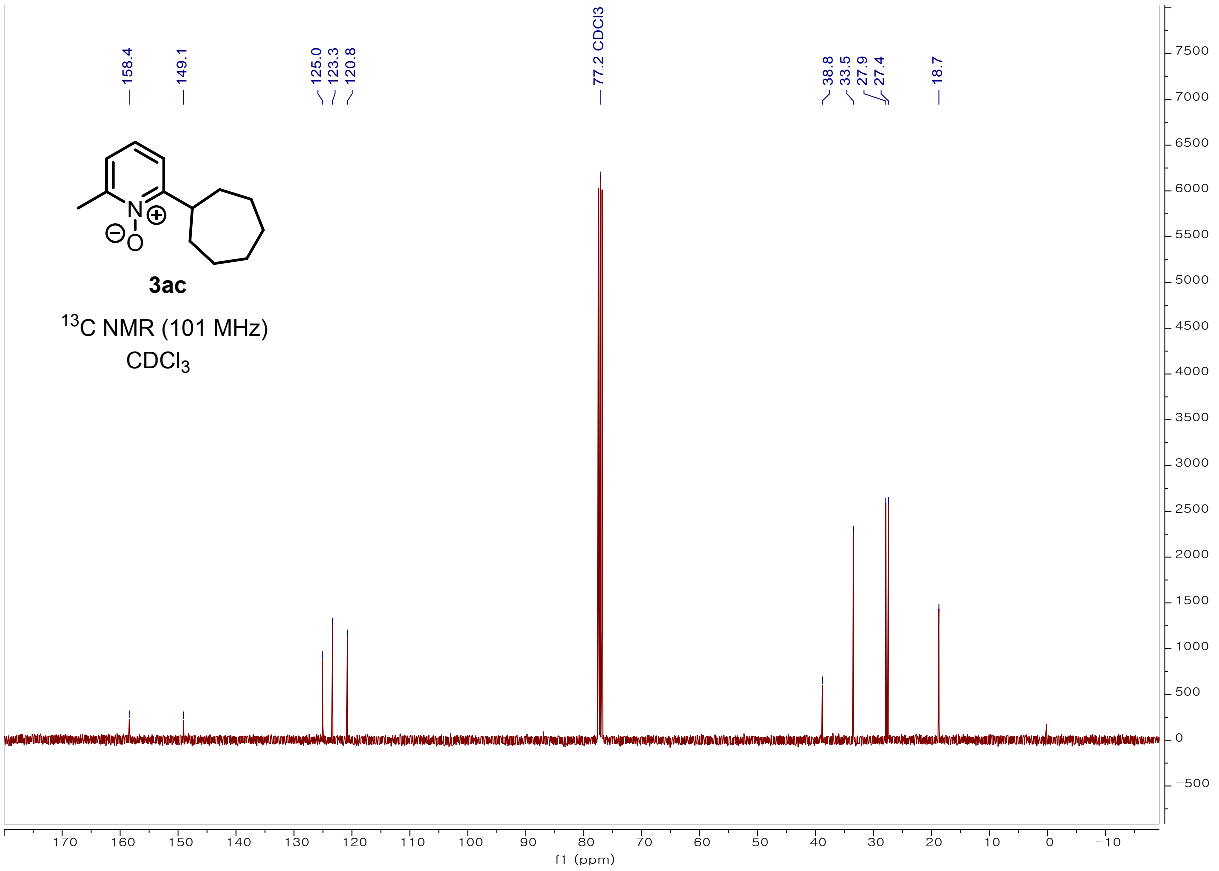

2-(1-(*tert*-butoxycarbonyl)piperidin-4-yl)-6-methylpyridine1-oxide (3ad)

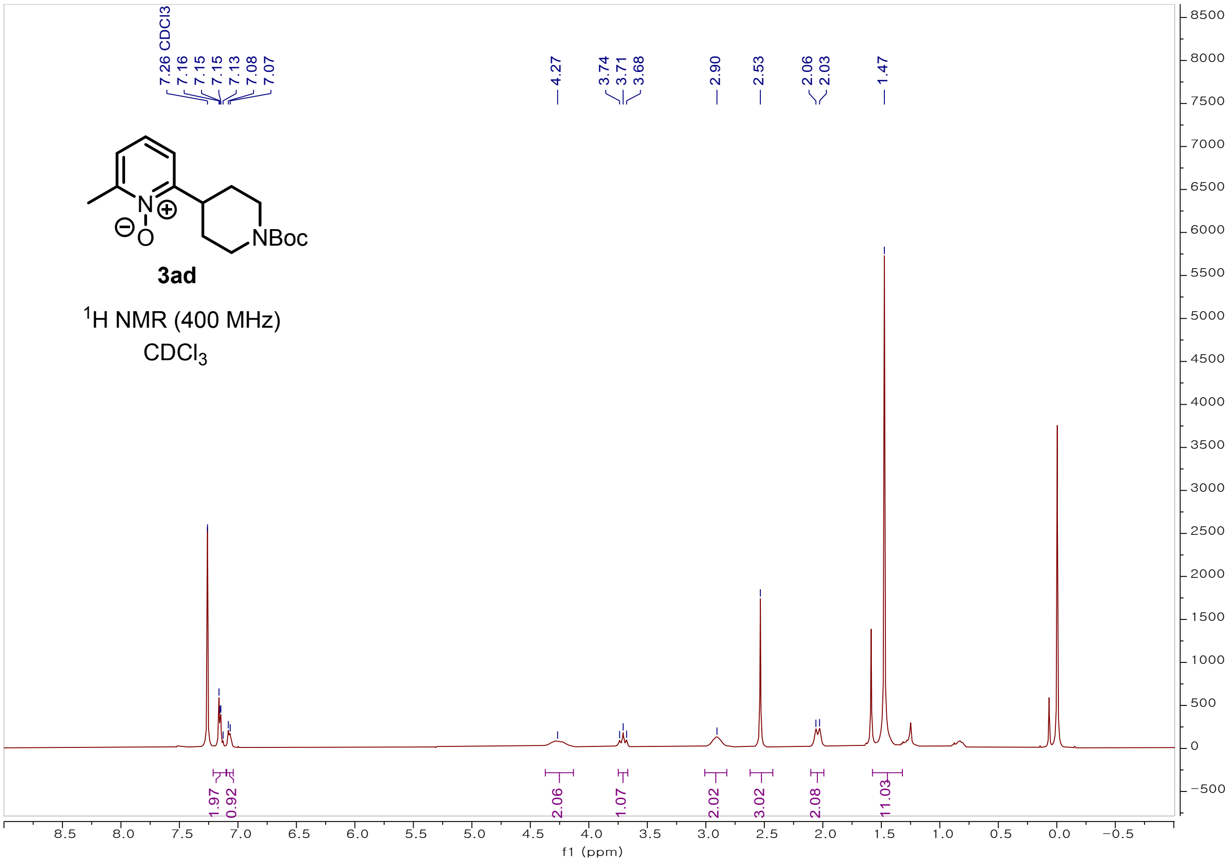

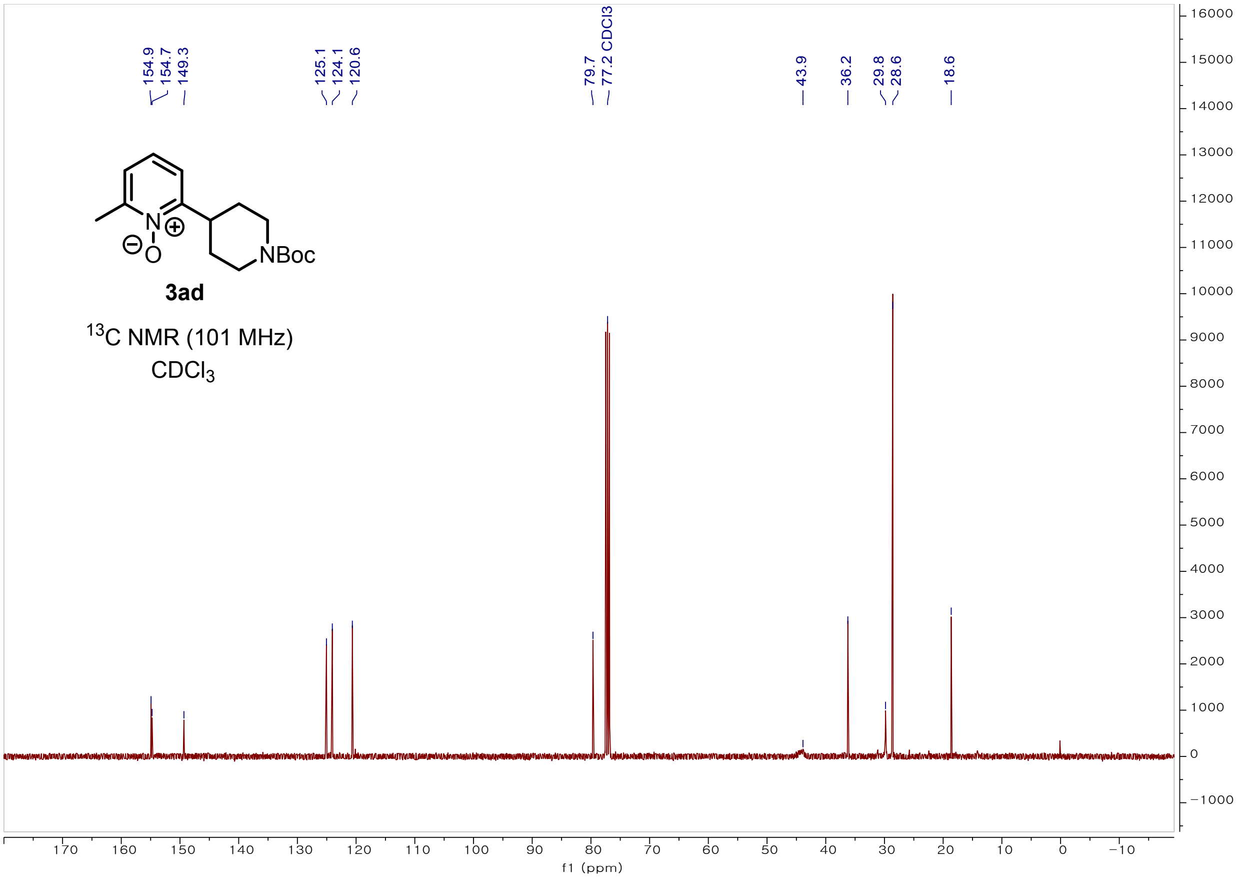

2-((1*S*,2*S*,4*R*)-bicyclo[2.2.1]heptan-2-yl)-6-methylpyridine 1-oxide (3ae)

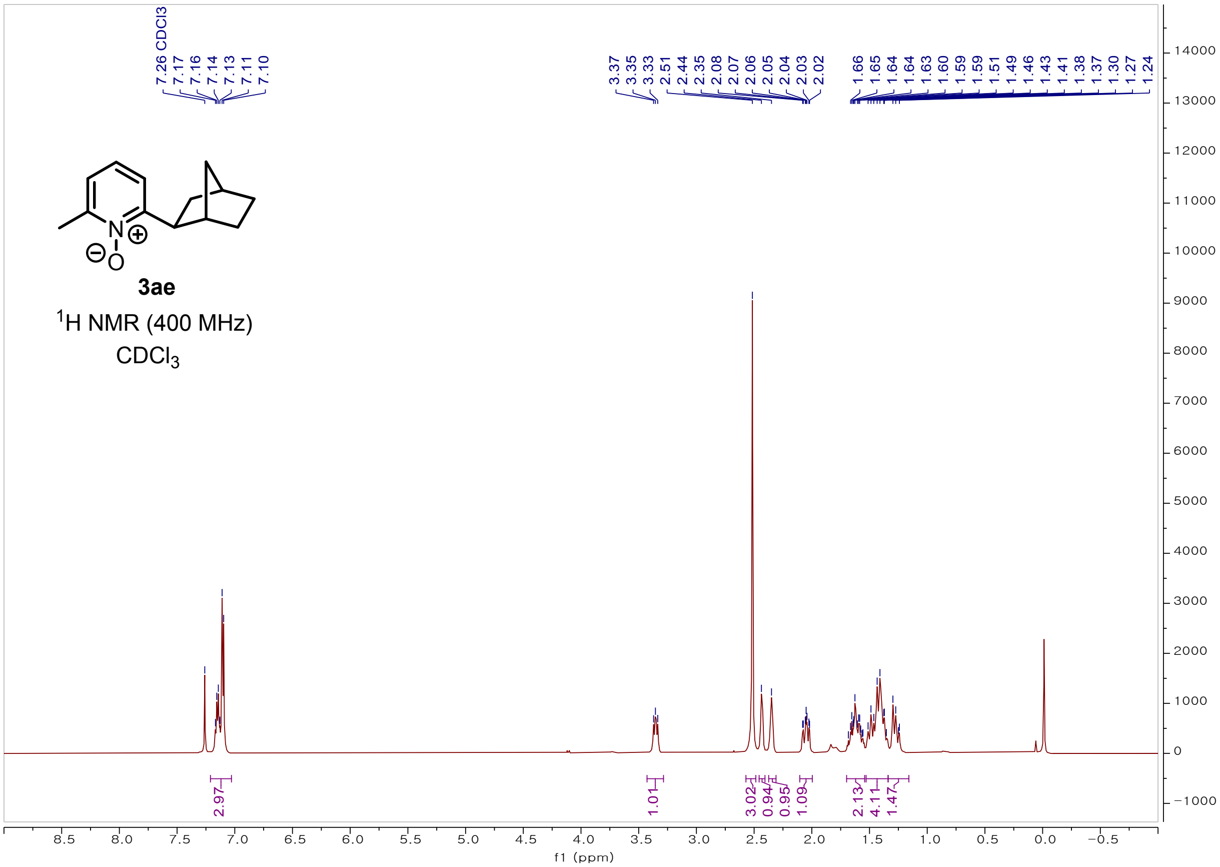

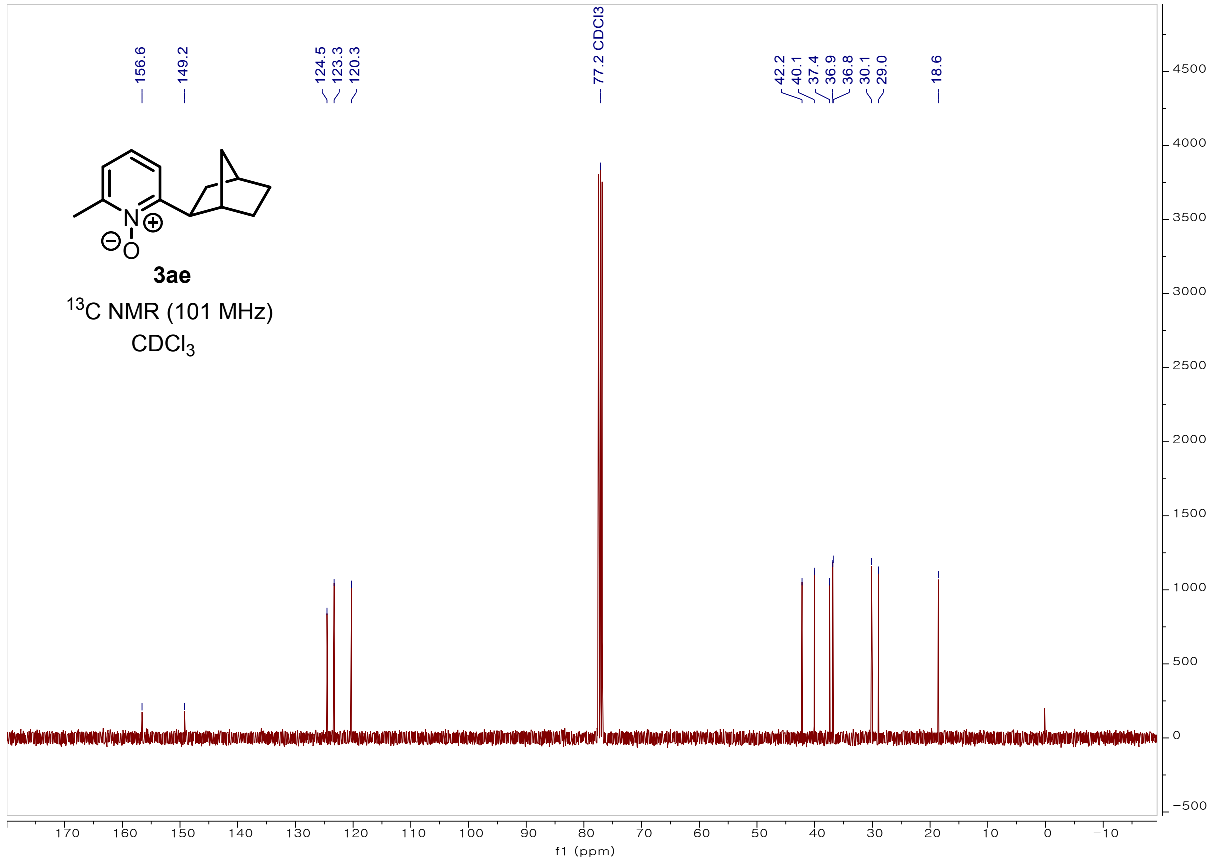

2-(*sec*-butyl)-6-methylpyridine 1-oxide (3af)

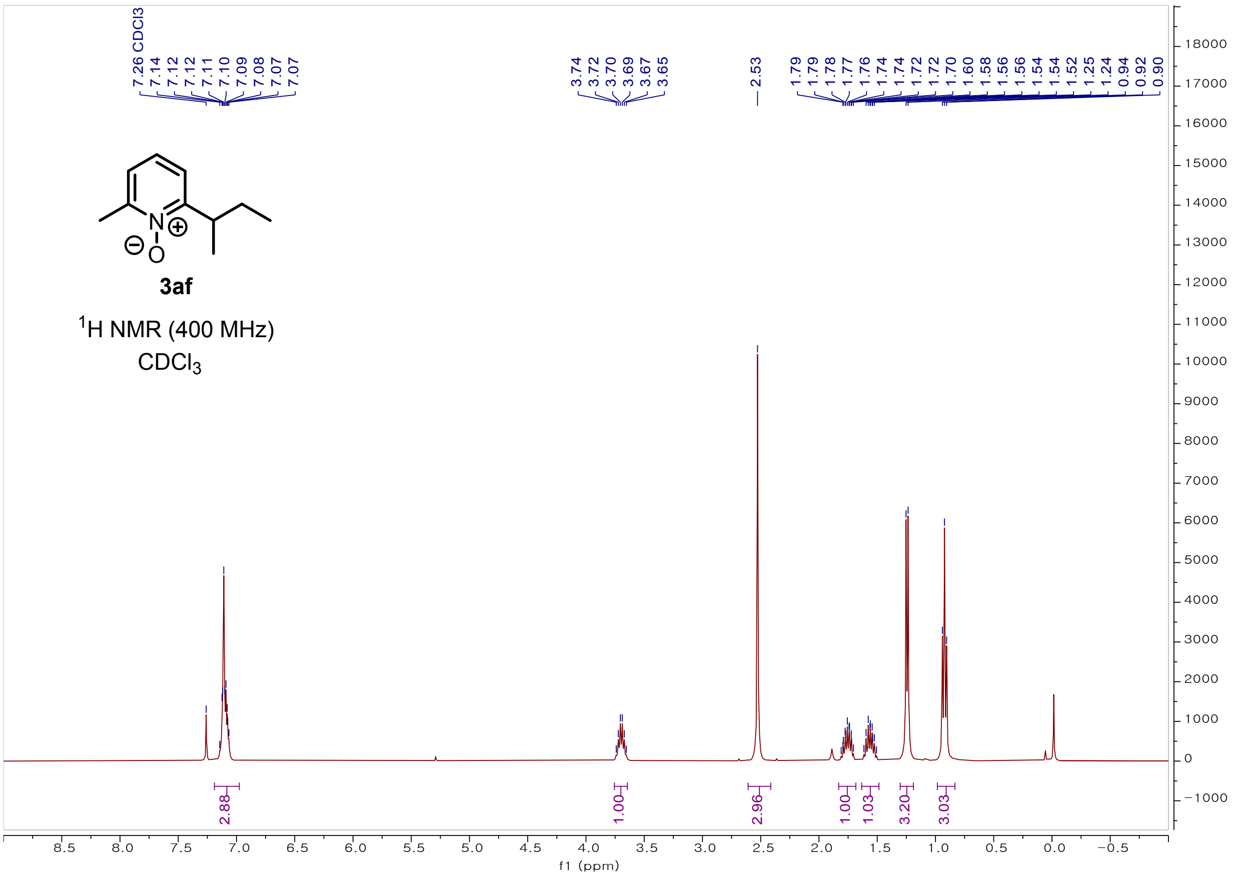

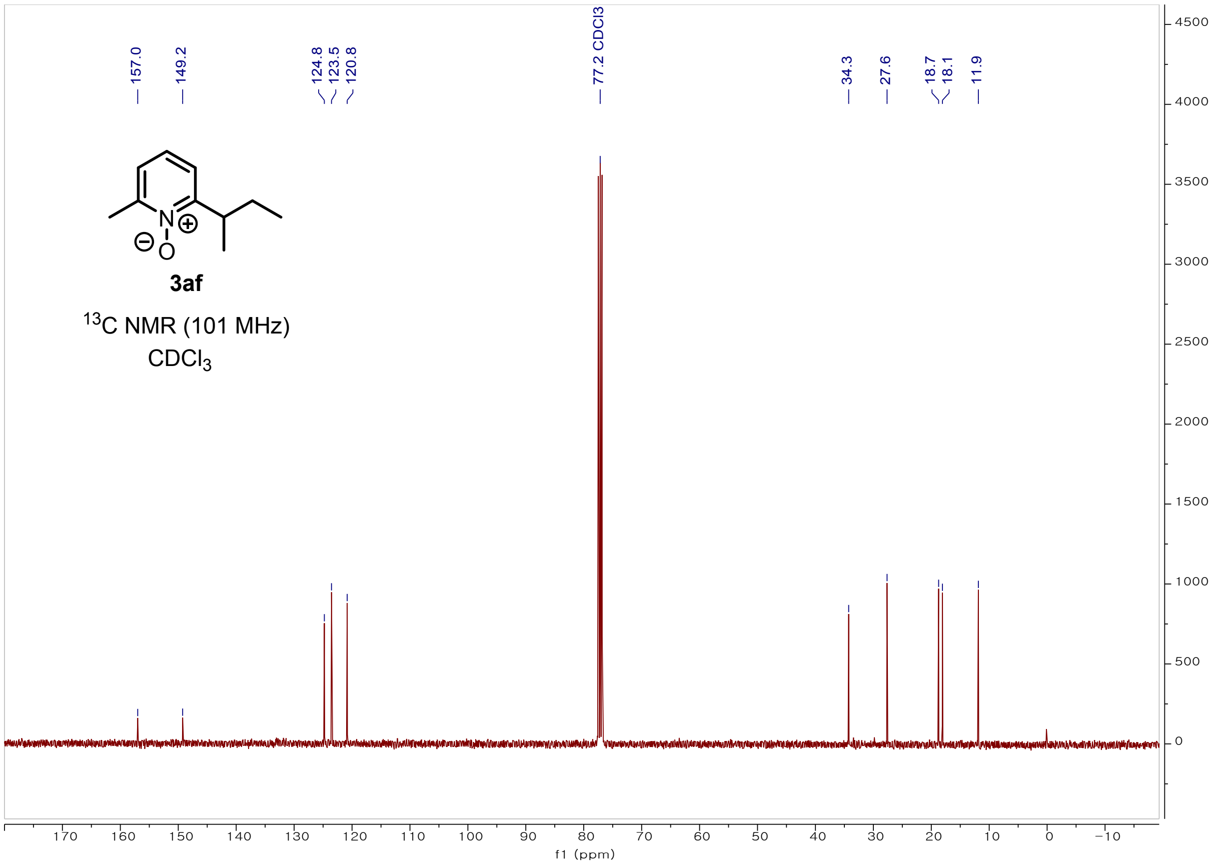

2-methyl-6-(pentan-3-yl)pyridine 1-oxide (3ag)

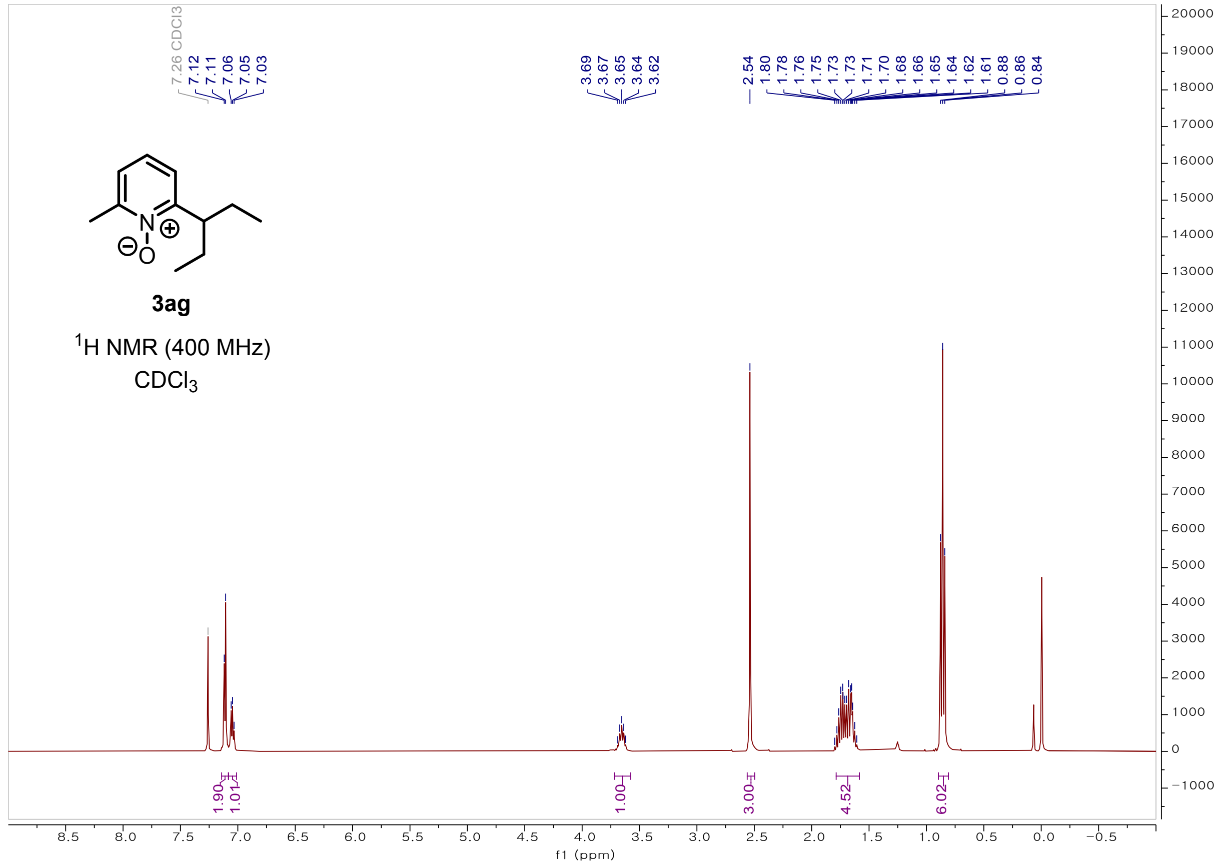

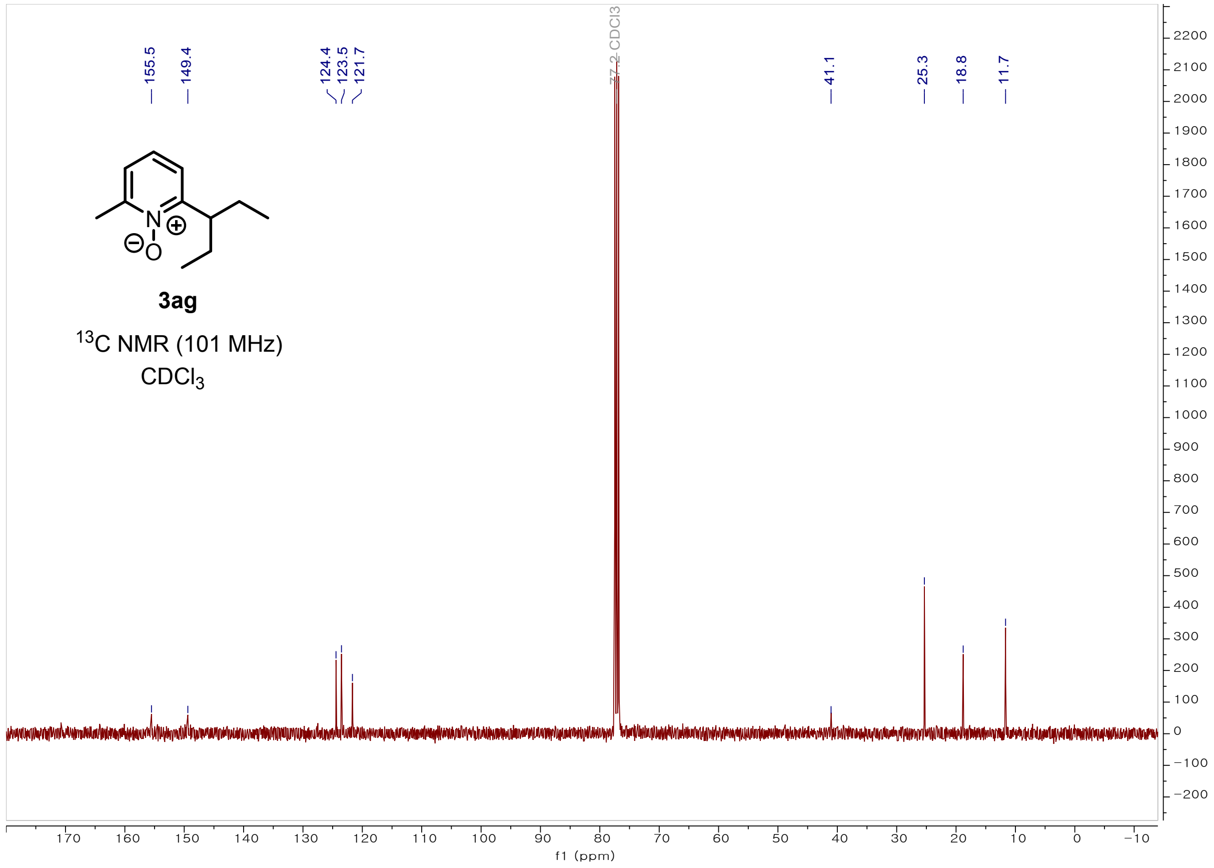

2-isobutyl-6-methylpyridine 1-oxide (3ah)

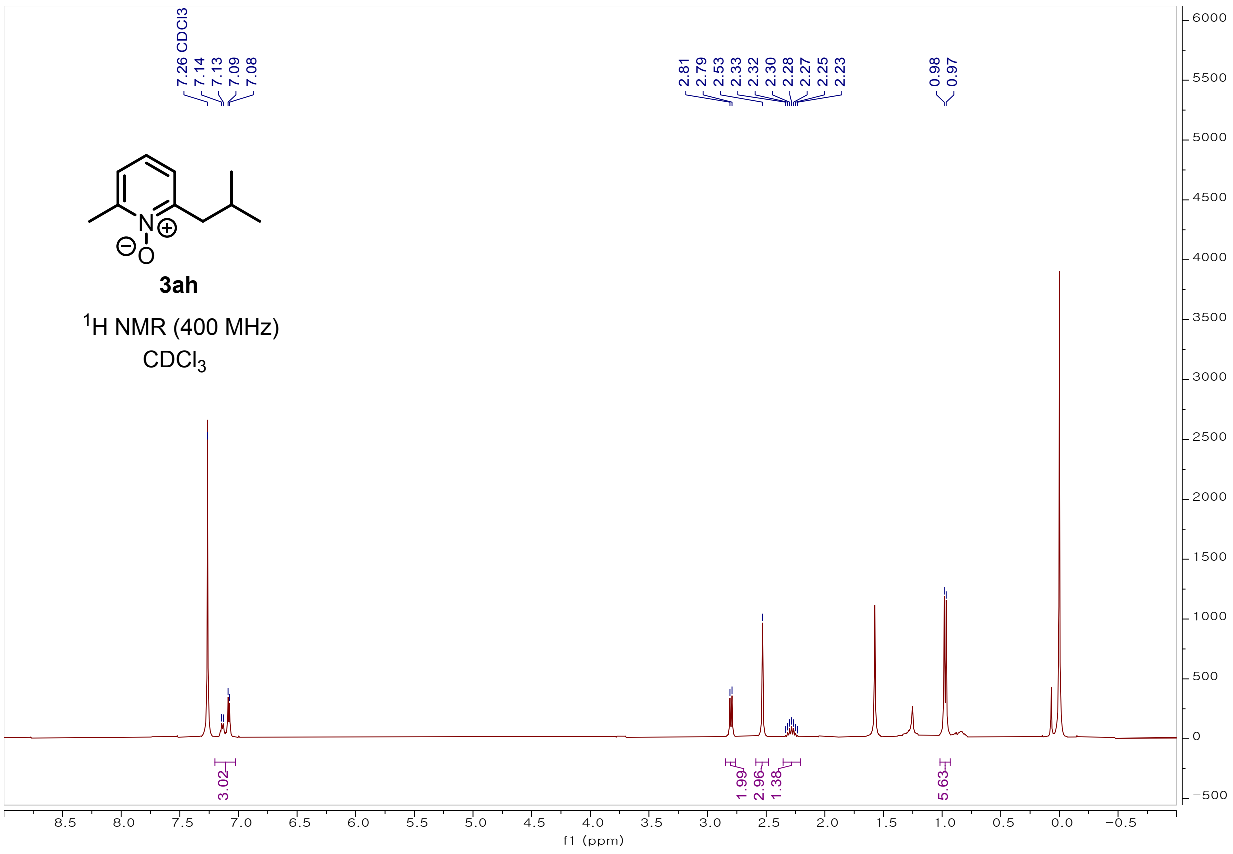

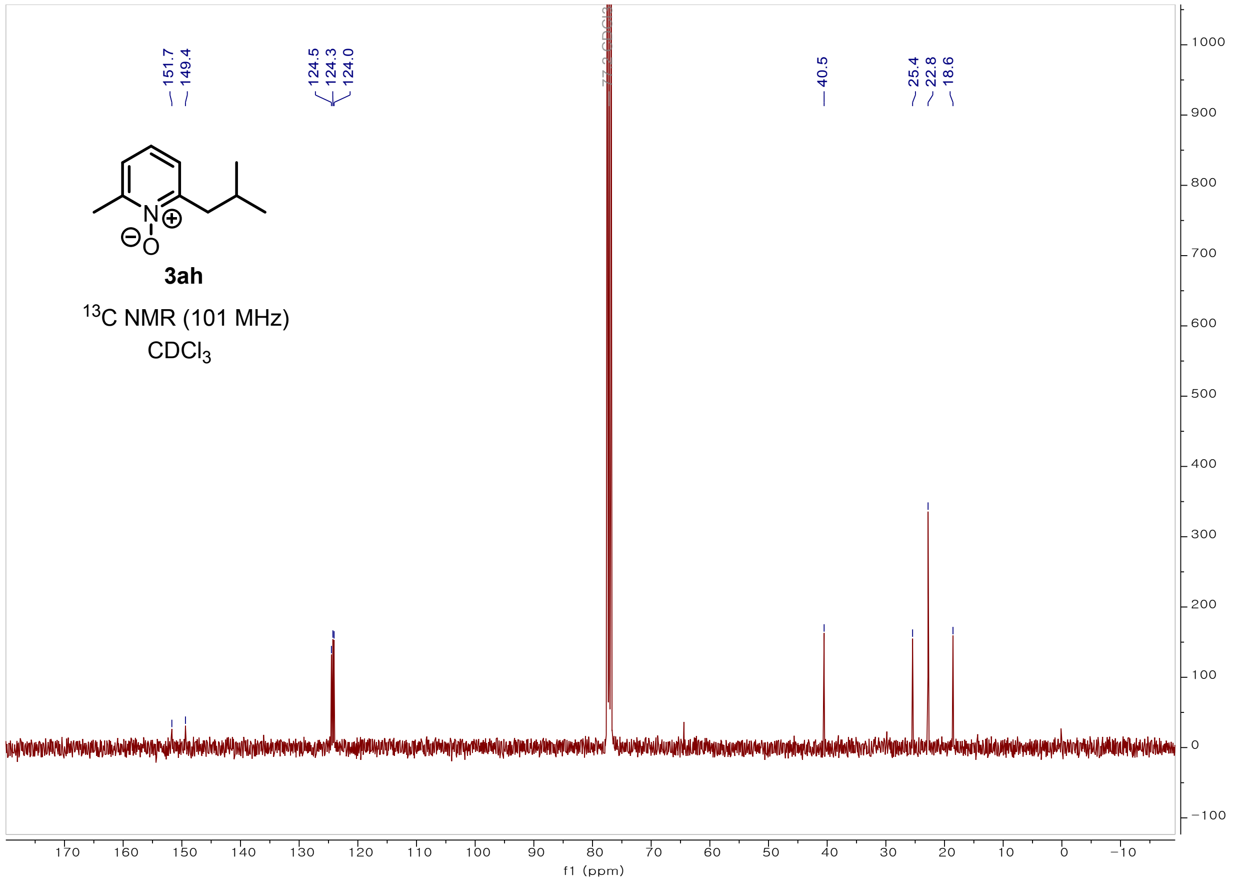

2-(*tert*-butyl)-6-methylpyridine 1-oxide (3ai)

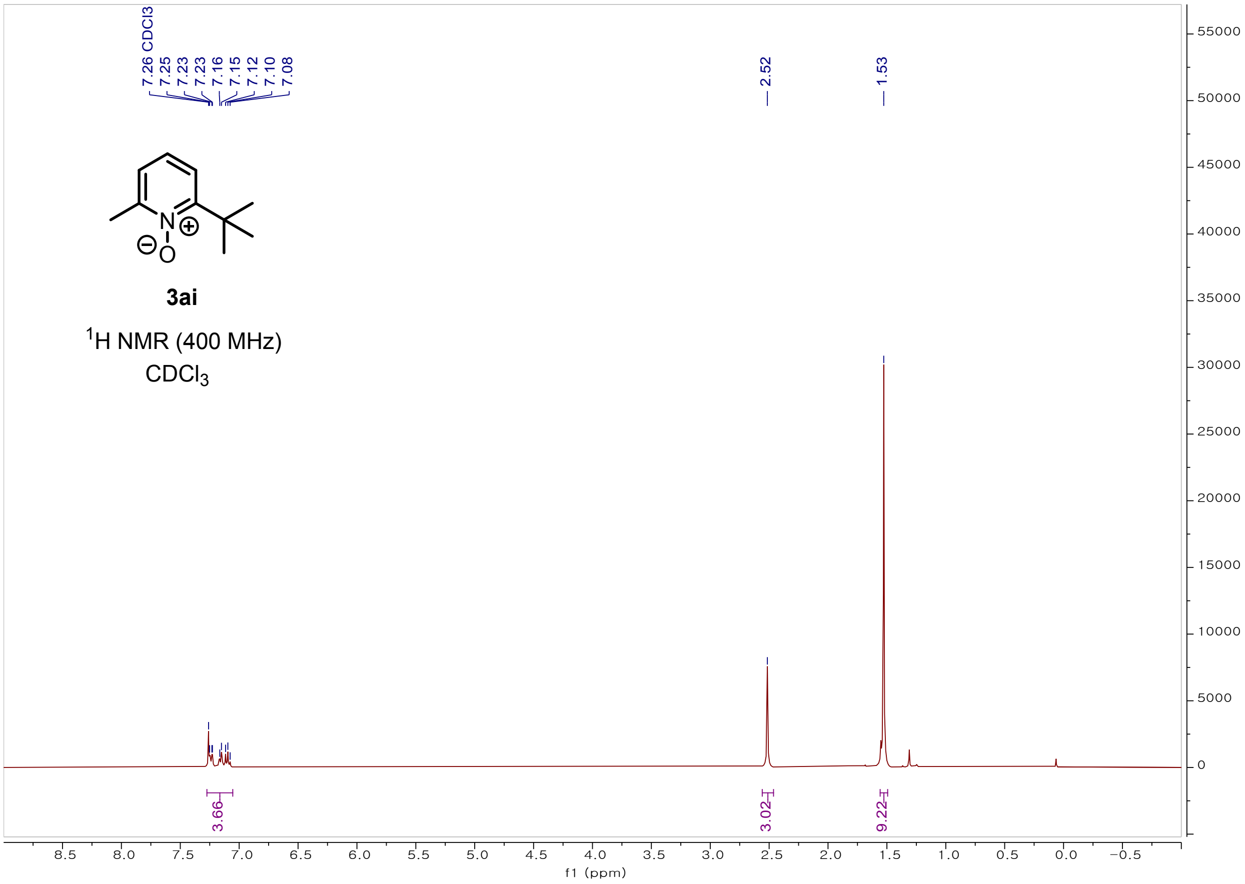

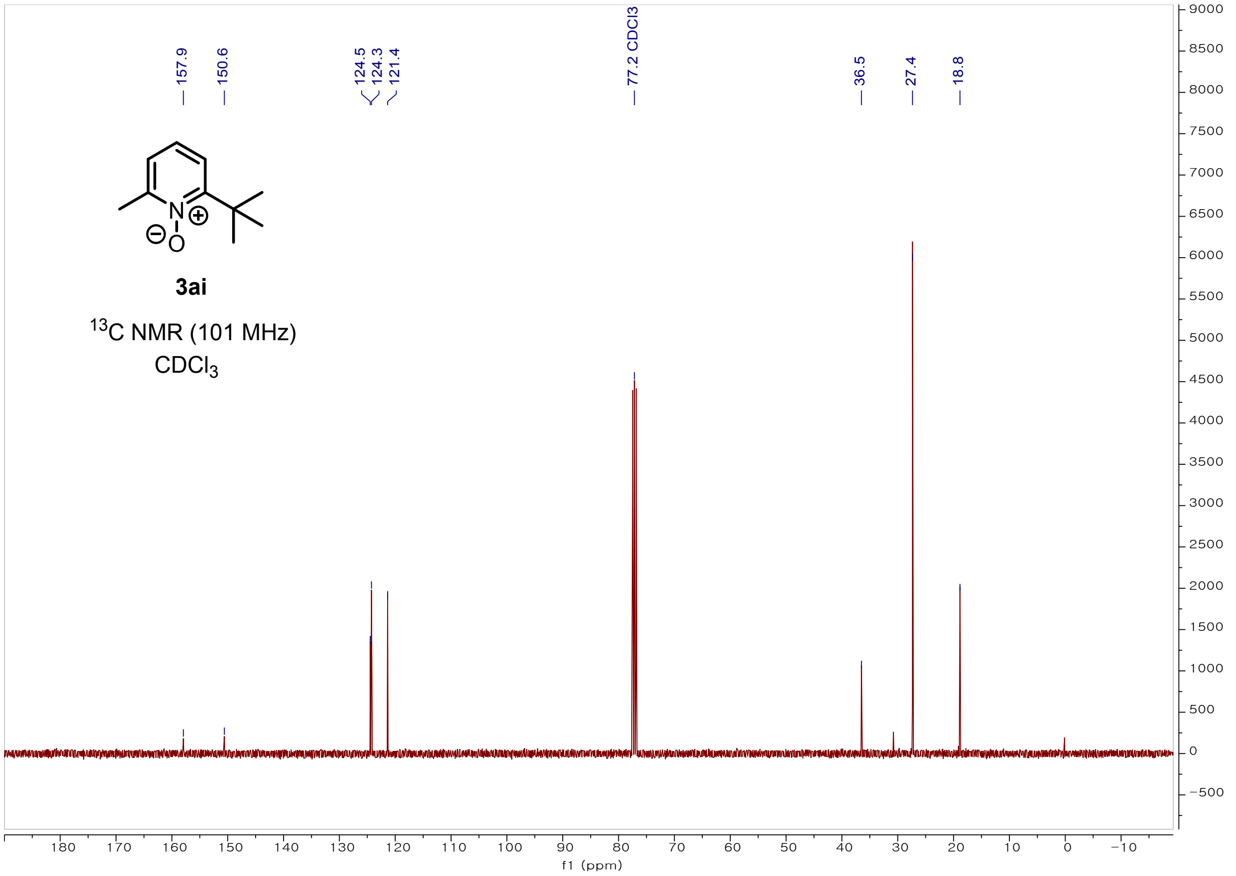

2-cyclohexyl-6-phenylpyridine 1-oxide (3ba)

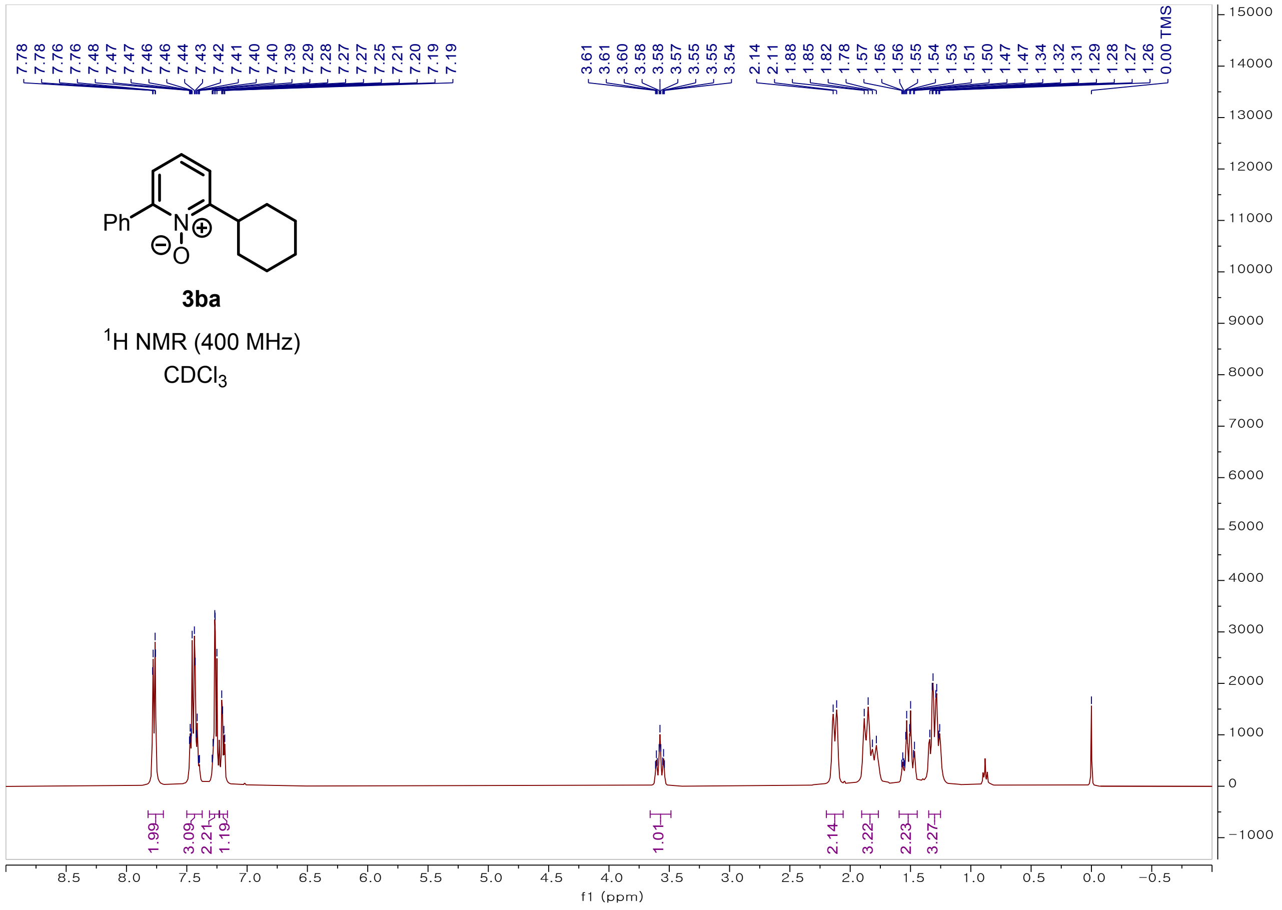

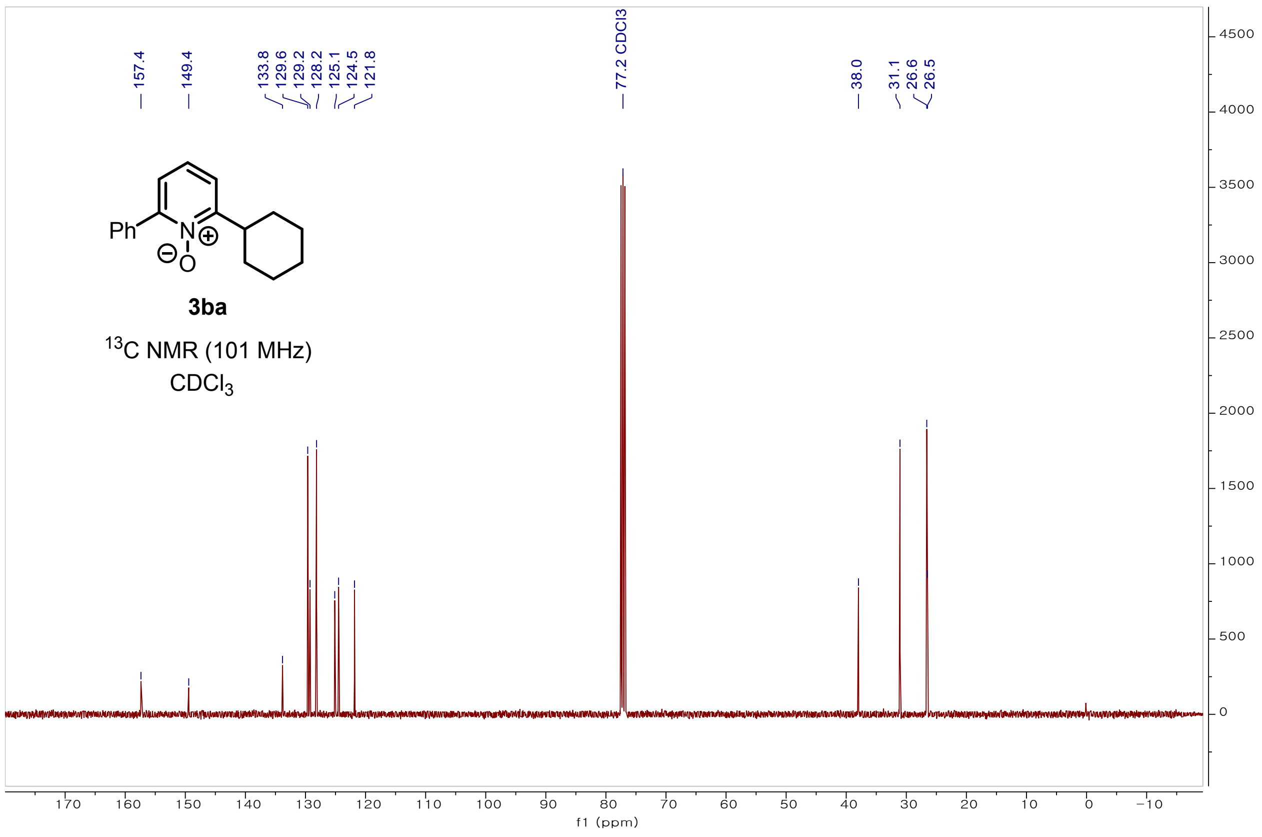

2-cyclohexylquinoline 1-oxide (3ca)

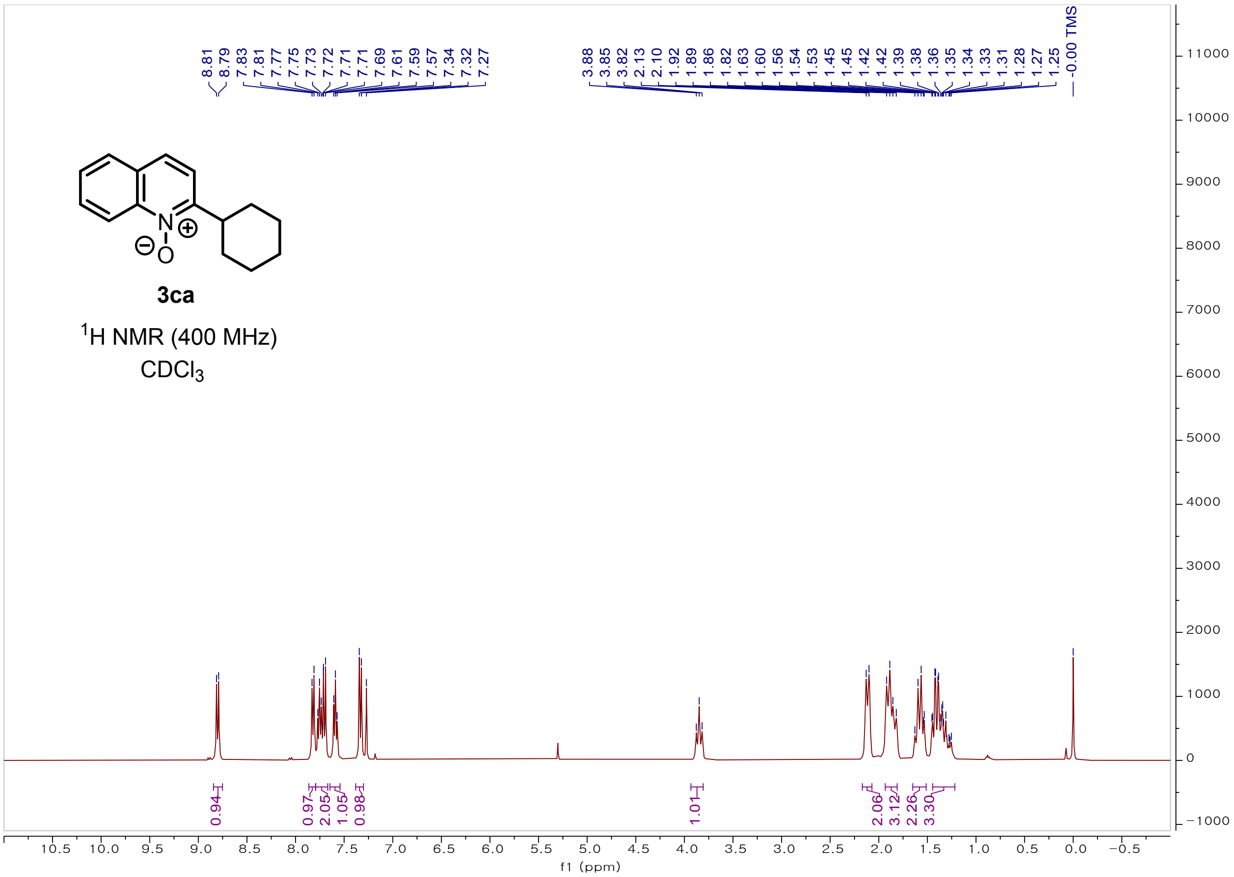

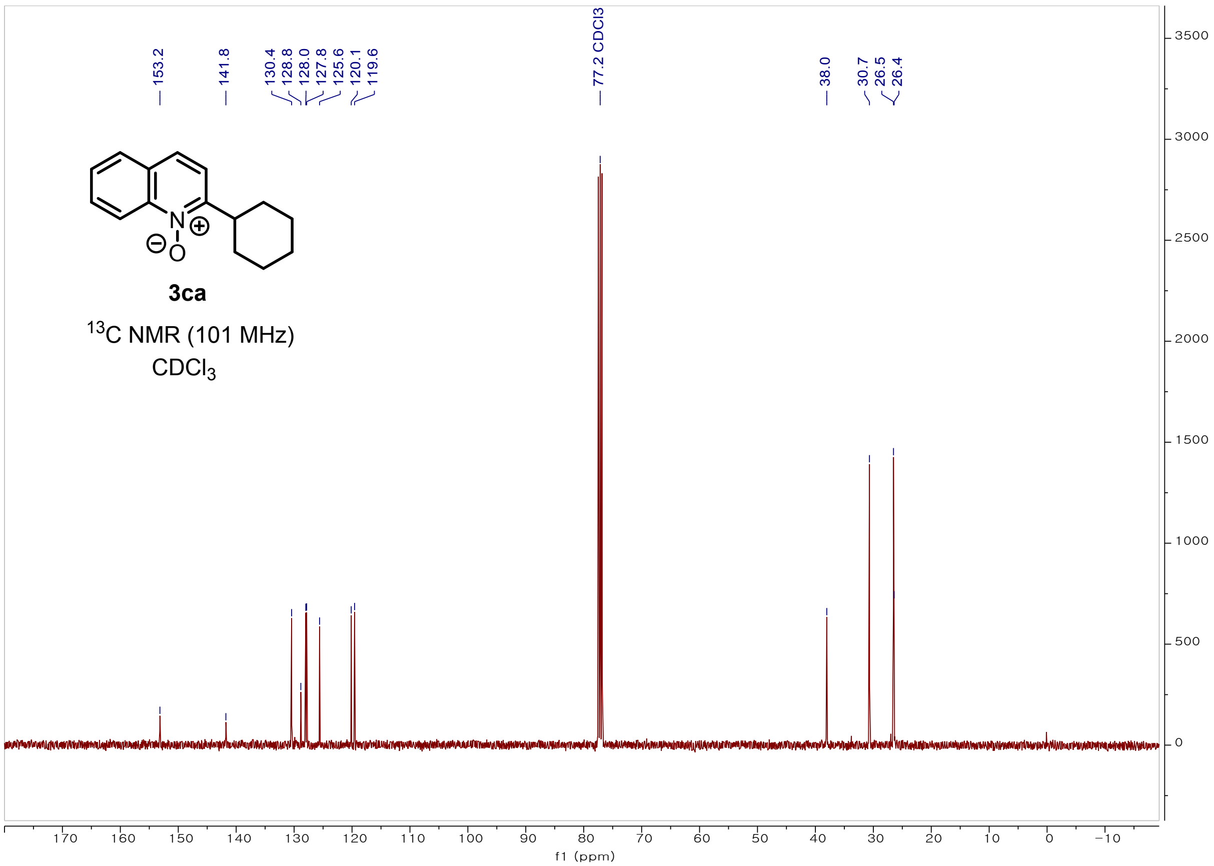

2-cyclohexyl-4-methylquinoline 1-oxide (3da)

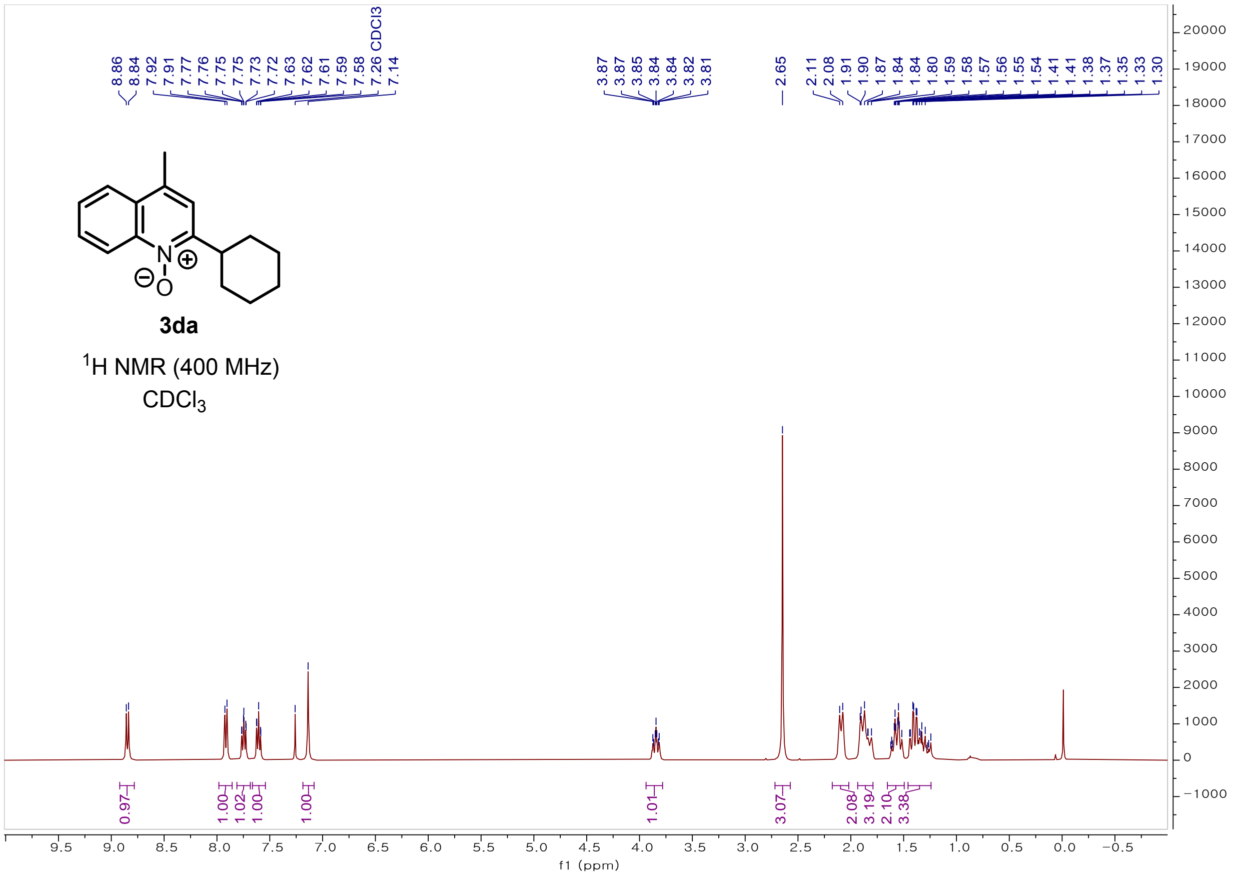

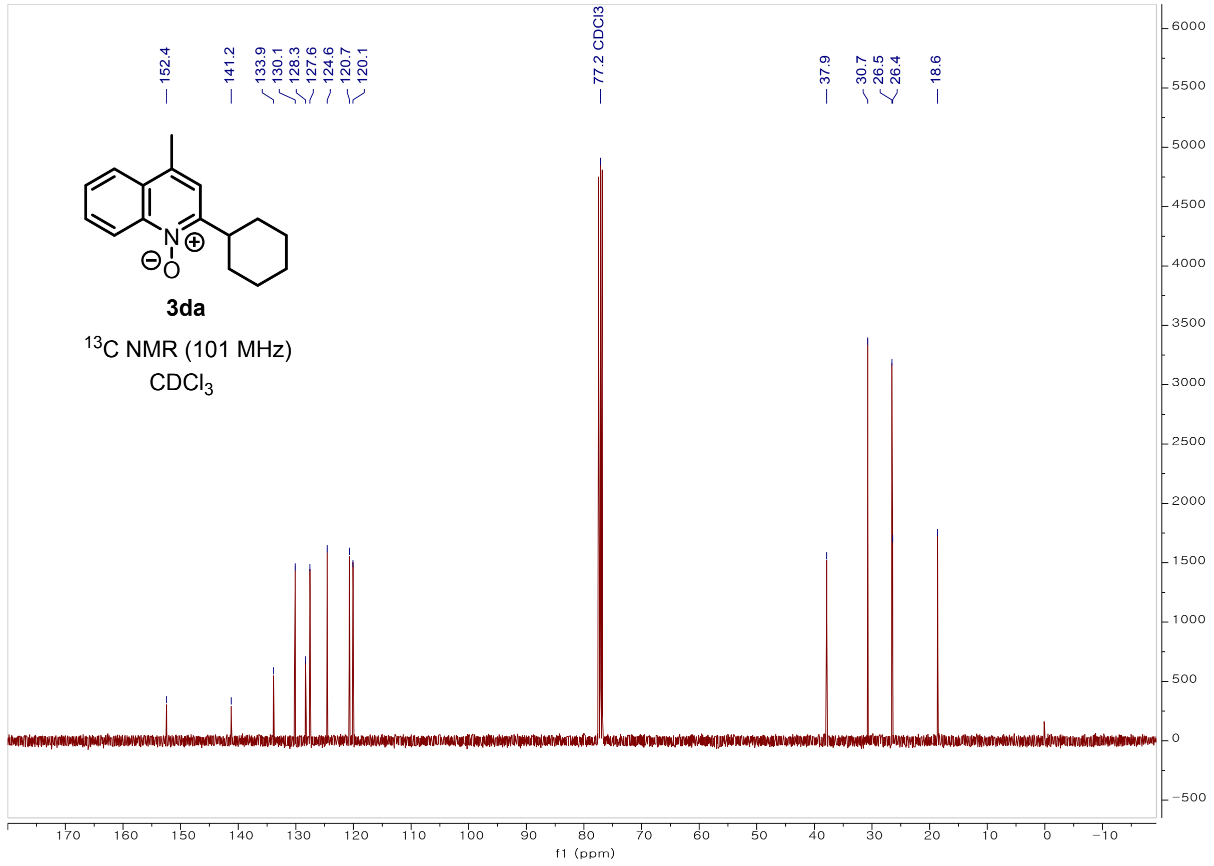

2-cyclohexyl-6-methylquinoline 1-oxide (3ea)

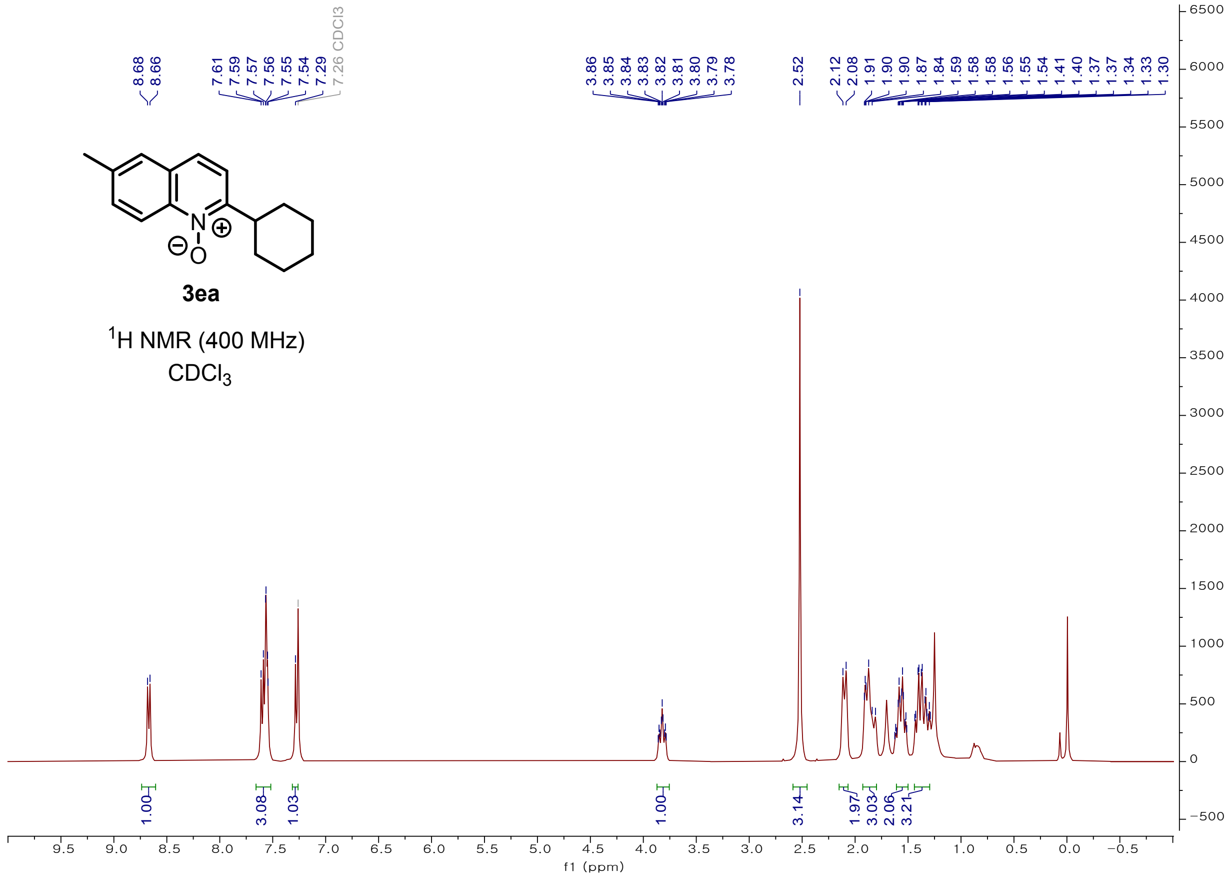

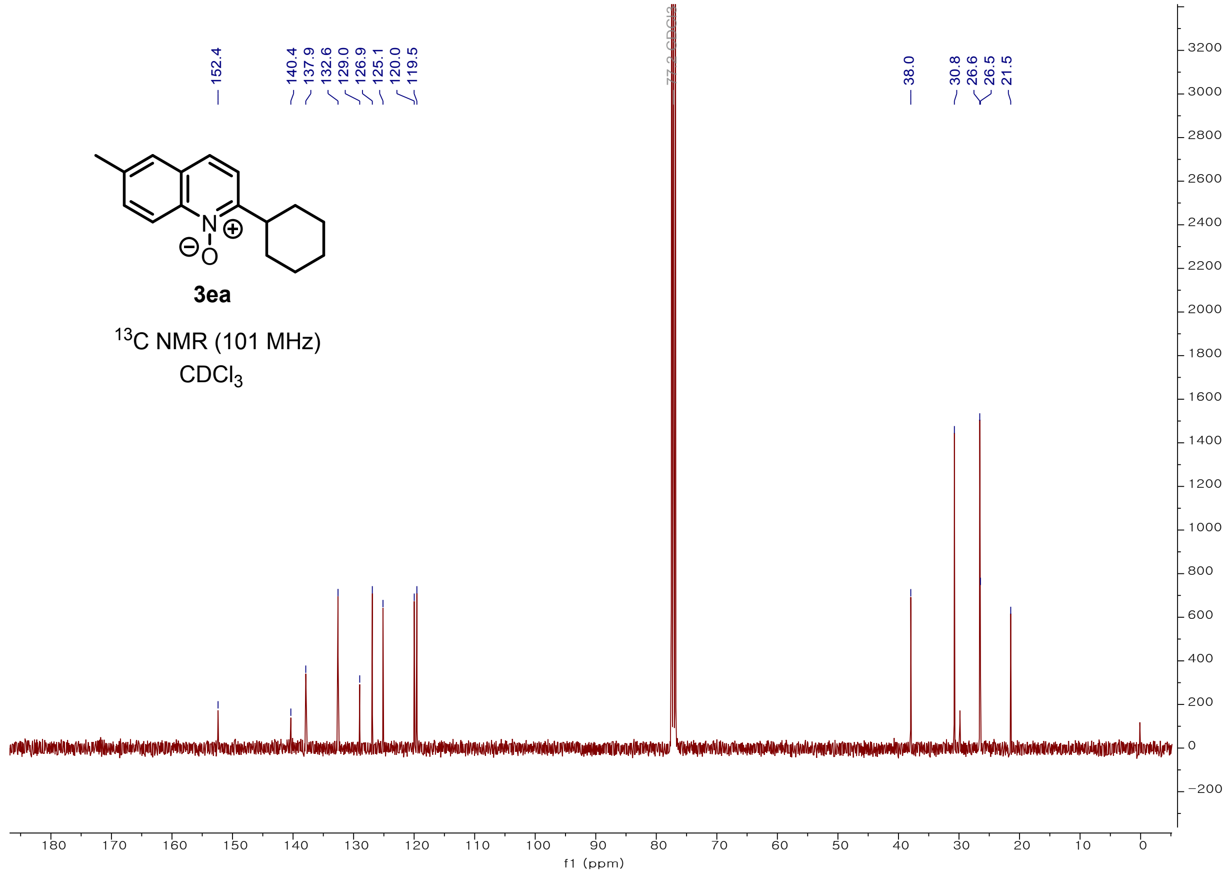

2-cyclohexyl-6-methoxynaphthalene (3fa)

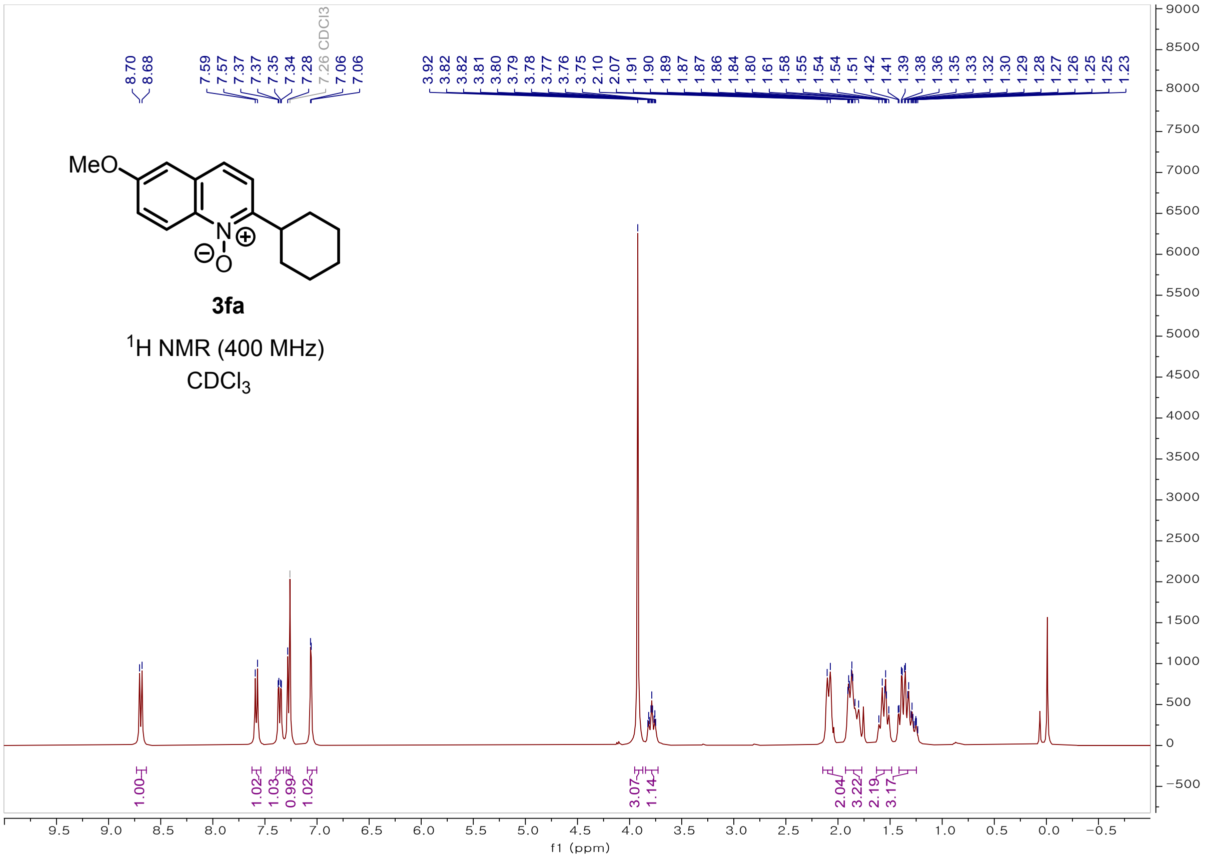

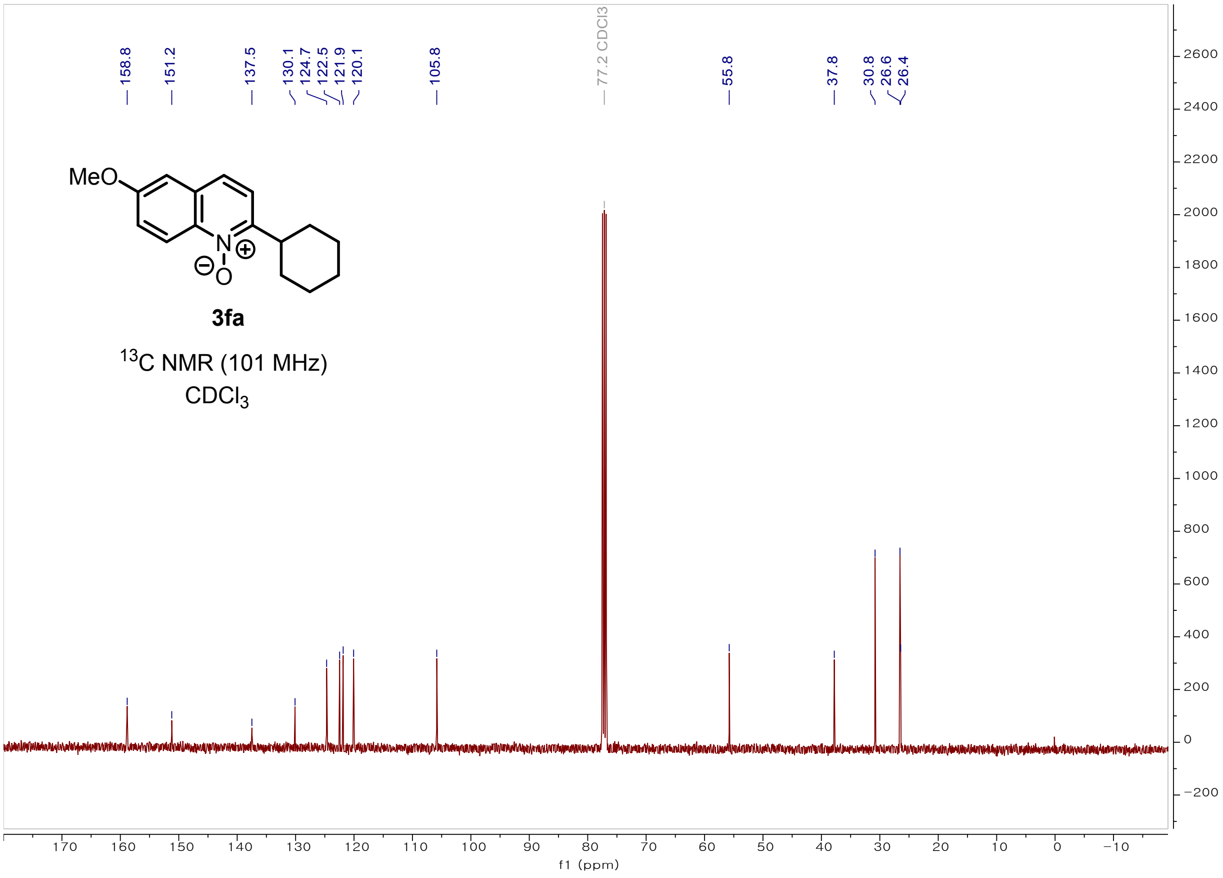

2-cyclohexylbenzo[*h*]quinoline 1-oxide (3ga)

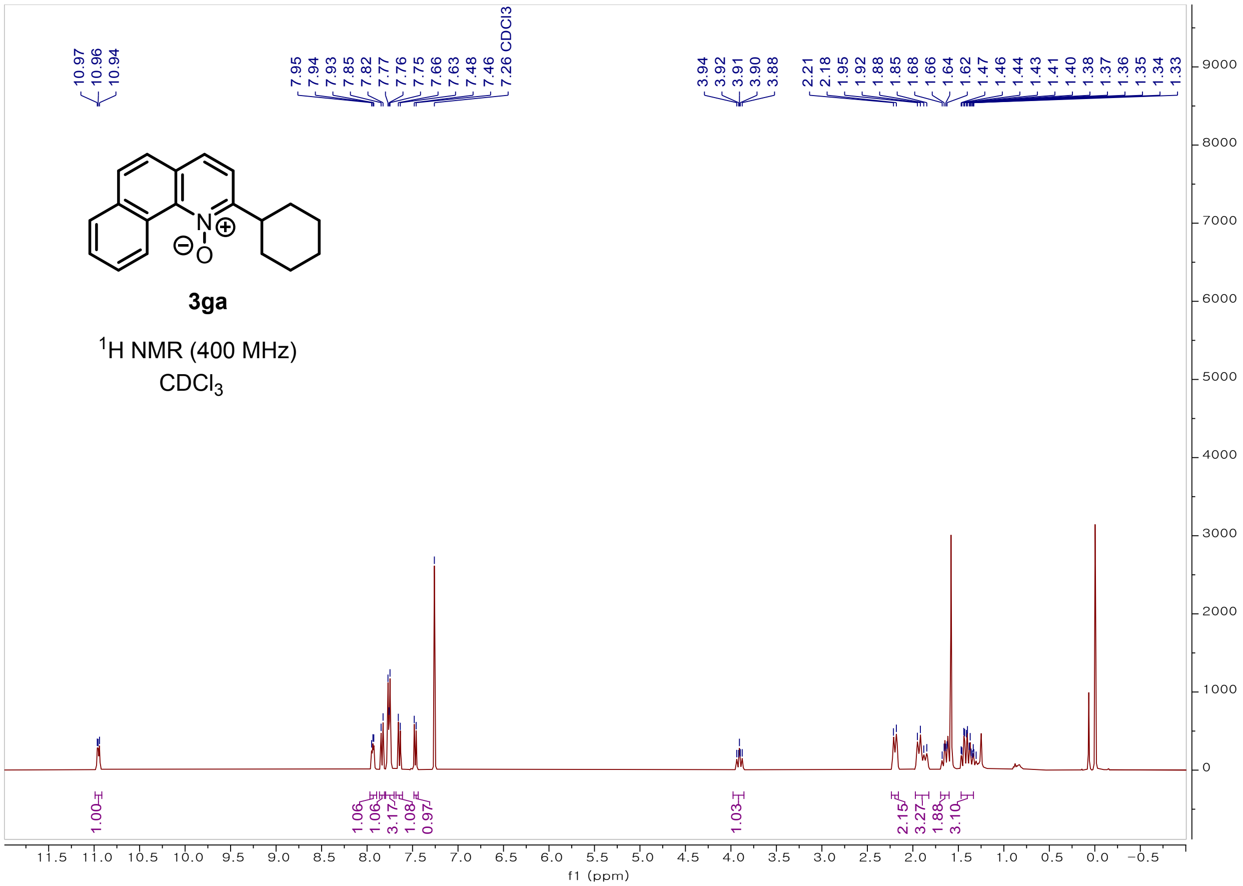
10.97
10.96
10.94
7.95
7.94
7.93
7.85
7.82
7.77
7.76
7.75
7.66
7.63
7.48
7.46
7.26 CDCl3
3.94
3.92
3.91
3.90
3.88
2.21
2.18
1.95
1.92
1.88
1.85
1.68
1.66
1.64
1.62
1.47
1.46
1.44
1.43
1.41
1.40
1.38
1.37
1.36
1.35
1.34
1.33
3ga
1H NMR (400 MHz)
CDCl3
1.00
1.06
1.06
3.17
1.08
0.97
1.03
2.15
3.27
1.88
3.10
f1 (ppm)

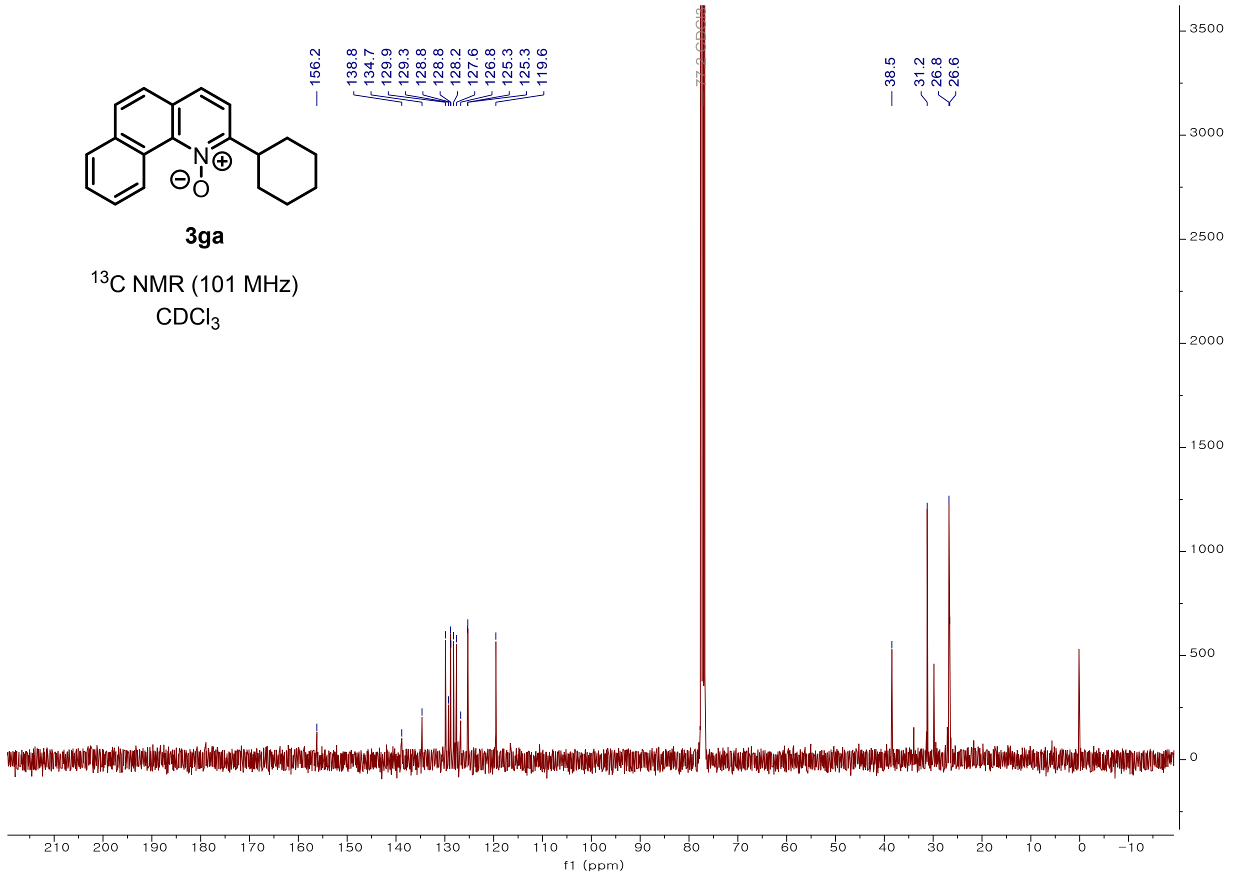
156.2
138.8
134.7
129.9
129.3
128.8
128.8
128.2
127.6
126.8
125.3
125.3
119.6
38.5
31.2
26.8
26.6
3ga
13C NMR (101 MHz)
CDCl3
f1 (ppm)
